# A Large-Scale Sequencing-Based Survey of Plasmids in *Listeria monocytogenes* Reveals Global Dissemination of Plasmids

**DOI:** 10.3389/fmicb.2021.653155

**Published:** 2021-03-12

**Authors:** Stephan Schmitz-Esser, Justin M. Anast, Bienvenido W. Cortes

**Affiliations:** ^1^Department of Animal Science, Iowa State University, Ames, IA, United States; ^2^Interdepartmental Microbiology Graduate Program, Iowa State University, Ames, IA, United States

**Keywords:** *Listeria monocytogenes*, plasmid, Tn7075, stress survival, genome

## Abstract

The food-borne pathogen *Listeria monocytogenes* is known for its capacity to cope with multiple stress conditions occurring in food and food production environments (FPEs). Plasmids can provide benefits to their host strains, and it is known that various *Listeria* strains contain plasmids. However, the current understanding of plasmid frequency and function in *L. monocytogenes* strains remains rather limited. To determine the presence of plasmids among *L. monocytogenes* strains and their potential contribution to stress survival, a comprehensive dataset was established based on 1,921 published genomes from strains representing 14 *L. monocytogenes* sequence types (STs). Our results show that an average of 54% of all *L. monocytogenes* strains in the dataset contained a putative plasmid. The presence of plasmids was highly variable between different STs. While some STs, such as ST1, ST2, and ST4, contained few plasmid-bearing strains (<15% of the strains per ST), other STs, such as ST121, ST5, ST8, ST3, and ST204, possessed a higher proportion of plasmid-bearing strains with plasmids found in >71% of the strains within each ST. Overall, the sizes of plasmids analyzed in this study ranged from 4 to 170 kbp with a median plasmid size of 61 kbp. We also identified two novel groups of putative *Listeria* plasmids based on the amino acid sequences of the plasmid replication protein, RepA. We show that highly conserved plasmids are shared among *Listeria* strains which have been isolated from around the world over the last few decades. To investigate the potential roles of plasmids, nine genes related to stress-response were selected for an assessment of their abundance and conservation among *L. monocytogenes* plasmids. The results demonstrated that these plasmid genes exhibited high sequence conservation but that their presence in plasmids was highly variable. Additionally, we identified a novel transposon, Tn*7075*, predicted to be involved in mercury-resistance. Here, we provide the largest plasmid survey of *L. monocytogenes* to date with a comprehensive examination of the distribution of plasmids among *L. monocytogenes* strains. Our results significantly increase our knowledge about the distribution, composition, and conservation of *L. monocytogenes* plasmids and suggest that plasmids are likely important for the survival of *L. monocytogenes* in food and FPEs.

## Introduction

The food-borne pathogen *Listeria monocytogenes* is a considerable public health concern because of its complex ecology involving environmental saprophytic, food-production environment-associated, and intracellular life cycles (Freitag et al., 2009). *L monocytogenes* is responsible for the food-borne disease listeriosis, which can be fatal, particularly for infants, pregnant women, and immunocompromised individuals (Allerberger and Wagner, 2010; Schlech, 2019). *L. monocytogenes* can tolerate a number of food-relevant stress conditions, including high salt concentrations and acidic pH, and is capable of growing at refrigeration temperatures (Bucur et al., 2018). Because of these stress survival capabilities, *L. monocytogenes* can persist for extended time periods in FPEs despite the regular rigorous cleaning procedures used to mitigate microbial contamination (Carpentier and Cerf, 2011; Ferreira et al., 2014). The persistence and survival of *L. monocytogenes* in FPEs represents a considerable challenge for food safety as it provides opportunities for *L. monocytogenes* to contaminate food. *L. monocytogenes* can be isolated from diverse food products, including ready-to-eat (RTE) foods which are at particular risk from a food safety perspective as they lack a heating step which would inactivate *L. monocytogenes*. Indeed, many listeriosis outbreaks have been linked to *L. monocytogenes* contamination of RTE foods including as deli meat, cheeses, fruits, and vegetables (Garner and Kathariou, 2016; Buchanan et al., 2017; Lopez-Valladares et al., 2018).

Extrachromosomal mobile genetic elements, such as plasmids, are important for rapid adaptation to changing environmental conditions. Plasmids can be transferred within and between bacterial species, lost by the host, or retained if they provide an advantage to survival. Plasmids can carry a variety of genes that may be beneficial for their host microorganisms under particular conditions, such as the presence of stressors, disinfectants, and antibiotics (Smalla et al., 2015). Previous studies have shown that plasmids can be found in many *L. monocytogenes* strains with up to 92% of the analyzed strains carrying plasmids. However, the frequency of plasmid carriage varies depending on the ST, serotype, and lineage (Kolstad et al., 1992; Lebrun et al., 1992; McLauchlin et al., 1997; Harvey and Gilmour, 2001; Hingston et al., 2017; Muhterem-Uyar et al., 2018; Chen et al., 2020; Palma et al., 2020). Other studies have also indicated that within specific STs, such as ST121, ST155, ST204, ST7, and ST87, *L. monocytogenes* strains can be found that carry virtually identical plasmids despite the strains having been isolated decades apart and/or originating from different countries (Schmitz-Esser et al., 2015b; Rychli et al., 2017; Wang et al., 2019; Palma et al., 2020).

Until recently, much of the *L. monocytogenes* plasmid research has focused on antibiotic resistance (Poyart-Salmeron et al., 1990; Hadorn et al., 1993; Charpentier et al., 1999; Li et al., 2016; Yan et al., 2020). Interestingly, other recent studies indicate that some *Listeria* plasmids may confer advantages to survival under stress conditions encountered in FPEs and food. These protections include increased tolerance to acidic, salt, oxidative, heat, and cold stress (Hingston et al., 2017, 2019; Pontinen et al., 2017; Naditz et al., 2019). Other plasmids provide increased tolerance to disinfectants, such as quaternary ammonium compounds (QACs) and heavy metals, allowing *L. monocytogenes* to tolerate higher levels of cleaners and detergents when exposed to sublethal concentrations of these compounds (Lebrun et al., 1994; Elhanafi et al., 2010; Xu et al., 2016; Kremer et al., 2017; Kropac et al., 2019). Currently, only one study has reported a possible link between *L. monocytogenes* plasmids and virulence. This plasmid, pLMIV, was isolated from the goat-derived *L. monocytogenes* strain FSL J1-208 and contains several genes belonging to the internalin family of virulence-associated *L. monocytogenes* proteins (den Bakker et al., 2012).

Previous research suggests that the rate of plasmid carriage is not equal across STs with some STs more likely to have plasmid-carrying strains than others. Additionally, plasmid-carrying strains may be more abundant than previously thought (Schmitz-Esser et al., 2015b; Hingston et al., 2017, 2019; Rychli et al., 2017). However, these earlier studies of plasmid prevalence in the genus *Listeria* were limited, as a comparatively low number of total genomes representing only a few STs of interest were analyzed. For example, Kuenne et al. (2010) conducted an analysis of 14 *Listeria* plasmids. Though the low number of *Listeria* genomes available at the time limited its sample size, this study revealed important genetic features of different *Listeria* plasmids such as putative heavy metal and stress resistance genes. More recent studies expanded the findings of Kuenne et al. (2010), performing in-depth sequence analyses of the plasmids found in additional sets of *L. monocytogenes* strains. In 2016, Fox et al. (2016) studied *L. monocytogenes* ST204 strains and found plasmids in 86.6% of the strains. A study conducted on 70 ST121 *L. monocytogenes* genomes observed that 81.4% of the strains harbored nearly identical plasmids (Rychli et al., 2017). Another study published in 2017 by Hingston et al. (2017) found that 55% of 166 strains from various *L. monocytogenes* STs contained a plasmid. A later study from the same group focused on a set of 93 *L. monocytogenes* strains and analyzed both their genetic content and the expression levels of their putative stress response genes during exposure to stress conditions (Hingston et al., 2019). More recently, 48% of 201 *L. monocytogenes* RTE strains were found to harbor plasmids (Chen et al., 2020). Today, thousands of assembled *L. monocytogenes* genomes are available in public databases with associated metadata for many of those genomes. We performed a plasmid survey in *L. monocytogenes* based on a much larger dataset than previous studies (Kuenne et al., 2010; Hingston et al., 2017, 2019; Chen et al., 2020) in order to provide an up-to-date overview on the distribution and genetic content of plasmids in *L. monocytogenes* and to gain more knowledge about the potential contribution of these plasmids to survival under stress conditions. Our dataset consists of 1,921 assembled *L. monocytogenes* genomes available in NCBI representing 14 different and highly abundant STs derived from 32 countries and multiple origins of isolation (i.e., food, clinical, and environment). The survey was conducted with a focus on the presence of plasmids, plasmid conservation between and within STs, and gene content, focusing on genes possibly important for survival in food and FPEs.

## Materials and Methods

### Strain Selection

We performed an in-depth literature search for studies describing *L. monocytogenes* genomes and with published metadata available for each genome. For this, we used the search term “*Listeria monocytogenes* genome” for a search on NCBI PubMed. The search was limited to publications from January 2010 to September 2019; this search resulted in 962 hits. Based on their title and abstract contents, 508 of these studies did not focus on *L. monocytogenes* and were therefore excluded. An additional 224 studies were excluded because they did not report *L. monocytogenes* genomes. Among the remaining 230 studies, we focused our search on publications with assembled genomes to reduce the computational and bioinformatic effort associated with assembling hundreds or thousands of genomes. In addition, we only selected studies including at least five strains. Based on the above criteria, 33 peer-reviewed research articles published before or during September 2019 were selected for this study (Supplementary Table 1). Importantly, studies were selected without *a priori* knowledge of plasmid harborage to reduce bias toward selecting for plasmid-carrying strains. Next, we identified STs of interest, focusing on *L. monocytogenes* STs which have been shown to be abundant in food and FPEs (Maury et al., 2016; Moura et al., 2016; Bergholz et al., 2018; Painset et al., 2019). To ensure a sufficient sample size for each ST of interest, we did not include any STs which were represented by less than 50 strains in our set of publications. 14 STs from our set of publications met the above criteria. These STs are among the most abundant STs in recent large-scale surveys and review publications (Henri et al., 2016; Moura et al., 2016; Bergholz et al., 2018; Painset et al., 2019) indicating that our dataset is adequately representative of *L. monocytogenes* diversity. For each study, all strains belonging to one of the 14 selected STs were added to our dataset. If the Multi-Locus Sequence Type (MLST) of the strains was unknown, the genome sequences were submitted to the Center for Genomic Epidemiology (CGE) website for MLST determination (Larsen et al., 2012). The final dataset included both closed and draft genomes. Details for all strains included in this study can be found in Supplementary Table 2.

### Dataset Generation, BLAST Analyses, and Plasmid Identification

Based on the literature search, information for all included STs and all the strains within each ST was consolidated in Microsoft Excel, listing strains, accession number, source, location, and year of isolation. Once sorted, the .fasta files for each strain were downloaded from NCBI. For each ST, a local nucleotide BLAST (BLASTn) database containing all contigs was created. BLASTn searches were conducted against the created ST databases using selected reference plasmid sequences as the query, requiring a minimum of 90% nucleotide identity. The initial query utilized the plasmid from strain N1-011a, an ST3 environmental strain from the United States collected in 2013 as a query against all STs. This reference plasmid was selected because it is among the largest known *Listeria* plasmids (148,959 bp) and contains homologs of many plasmid-specific genes found among various STs. For further confirmation, additional BLASTn searches of all STs were performed; queries consisted of reference plasmids from various sources and with different plasmid replication protein RepA groups (Supplementary Table 3). RepA sequences have been used previously for plasmid classification in *L. monocytogenes* (Kuenne et al., 2010).

BLASTn results were obtained as tab-separated files. These were manually reviewed for each ST and filtered for BLASTn comparison matches with an alignment length of ≥500 bp and a minimum nucleotide sequence identity of 90%. Those identified contigs were then considered as potential plasmid contigs. As our database contained many draft genomes, many plasmids were assembled into multiple contigs; thus, for some strains, a putative plasmid consisted of multiple contigs. Hits to larger (>100 kbp) chromosomal contigs indicative of an integration of entire plasmids or plasmid segments into the chromosome were manually reviewed for their annotation using the PATRIC database (Davis et al., 2020) and excluded from further analyses. Additionally, the presence of a homolog to RepA on one of the putative plasmid contigs for a given strain was required for the strain to be considered as harboring a putative plasmid. To identify RepA homologs, the nucleotide and amino acid sequences representing a group 2 RepA (from pLM80 *L. monocytogenes* H7858, GenBank accession number ZP_00231652.1) and a group 1 RepA (from pLM33 *L. monocytogenes*, YP_003727990.1) (Kuenne et al., 2010) were used as query sequences for BLASTn and BLASTp searches. We used both group 1 and group 2 RepA representative sequences as queries in order to reliably identify diverse RepA proteins. Indeed, group 1 and group 2 RepA proteins share only 61% amino acid identity but are highly conserved within each RepA group. For plasmid replication protein typing, the plasmids were searched against the PLSDB plasmid database (Galata et al., 2019). In addition to the group 1 and 2 plasmids defined by their RepA sequence, *Listeria* species can also harbor smaller plasmids. Thus, as additional query sequences, we utilized the nucleotide and amino acid sequences of the plasmid replication and mobilization proteins identified on the small *L. monocytogenes* plasmids pLMST6 and pIP823 (Charpentier et al., 1999; Kremer et al., 2017). In contrast to group 1 and group 2 plasmids, which are theta-replicating plasmids (Kuenne et al., 2010), the small *Listeria* plasmids pLMST6 or pIP823 replicate by a rolling-circle mechanism and have replication proteins that show no similarity to the RepA proteins found in the group 1 and 2 *Listeria* plasmids. To further demonstrate that the potential plasmid contigs identified using BLAST indeed represented putative plasmids, selected genomes representing different abundant plasmid subtypes for each ST (based on the BLASTn results, see below) were uploaded to the PATRIC database for annotation and manual verification (Davis et al., 2020) as well as searched against the PLSDB database (Galata et al., 2019).

### Gene Content Analysis and Gene Conservation

The gene content of select plasmids was analyzed by manually reviewing the PATRIC annotations of the identified candidate plasmid contigs. Contigs were searched for the presence of genes encoding homologs to known plasmid-encoded proteins such as RepA, the heavy metal resistance proteins CadAC, and the stress response proteins ClpL and Tmr. Previous studies have demonstrated that *Listeria* plasmids possess a modular structure and very high levels of conservation (Kuenne et al., 2010; Schmitz-Esser et al., 2015b; Fagerlund et al., 2016; Fox et al., 2016; Hingston et al., 2017; Rychli et al., 2017). Thus, the conservation between different plasmids within and between STs was determined based on the BLASTn results considering alignment length (requiring at least 90% coverage of the query in the subject) and percent nucleotide identity. Based on these results, plasmids sharing >99.9% nucleotide identity and >99% coverage were considered plasmid subtypes. Alignments of plasmids were done with MAUVE (Darling et al., 2010) to compare different plasmid subtypes. Finally, after reviewing the published literature on *Listeria* plasmids, nine plasmid-encoded genetic elements involved in stress response were identified and used as query sequences for BLASTn searches to determine their presence on the plasmids identified in this study (Table 1).

**TABLE 1:** Selected plasmid-encoded genes of interest involved in stress response.

Plasmid stress response genes	Proposed/demonstrated function	References
Cadmium resistance ATPase c*adA1* (Tn*5422*)	Cadmium detoxification and resistance	Parsons et al., 2018
Cadmium resistance ATPase c*adA2* (pLM80)	Cadmium detoxification and resistance	Parsons et al., 2018
Triphenylmethane reductase *tmr* (pLM80)	Dye detoxification	Dutta et al., 2014
Heat-shock protease *clpL* (pLM58)	Increased heat stress tolerance. Upregulated under salt and acid stress	Pontinen et al., 2017; Hingston et al., 2019; Cortes et al., 2020
Benzalkonium chloride resistance cassette *bcrABC* (pLM80)	Increased quaternary ammonia compound (benzalkonium chloride) tolerance	Elhanafi et al., 2010
Multicopper oxidase *mco*	Copper homeostasis; mediates oxidative stress tolerance in *Staphylococcus aureus.* Upregulated under acid stress	Sitthisak et al., 2005; Hingston et al., 2019; Cortes et al., 2020
Putative NADH peroxidase *npr*	Mitigation of oxidative stress. Upregulated under salt and acid stress	Hingston et al., 2019
Uncharacterized NiCo riboswitch	Intergenic region linked to heavy metal binding. Upregulated under acid stress	Cortes et al., 2020
Putative glycine-betaine transporter binding protein *gbuC*	Involved in salt stress response. Upregulated under salt stress	Hingston et al., 2019

## Results

### Overview of the Dataset

**FIGURE 1:**
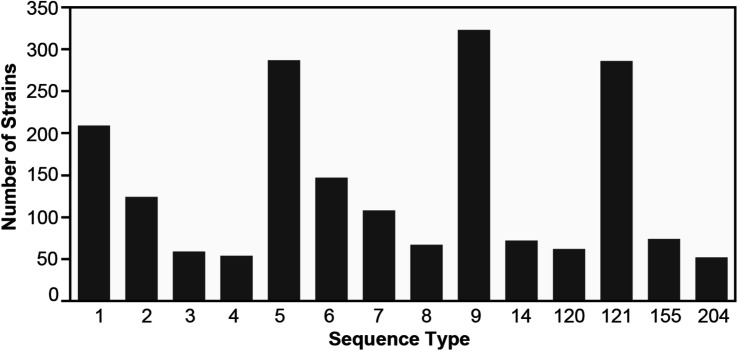
The number of *Listeria monocytogenes* genomes per ST included in this study. Supplementary Table 2 contains additional details on the strains.

**FIGURE 2:**
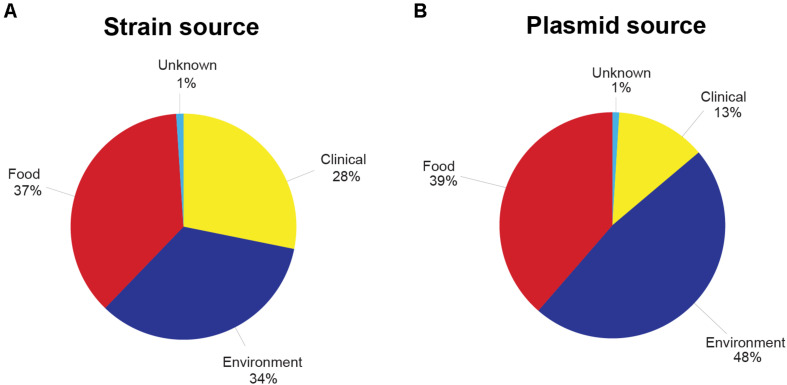
**(A)** Percentage of *Listeria monocytogenes* strains used in this study from each source of origin. **(B)** Percentage of putative *Listeria monocytogenes* plasmids from each source of origin. Clinical sources included human or animal clinical samples, whereas food sources were isolated from food products. Environmental sources included, but were not limited to, production facilities, environmental swabs, and equipment. See Supplementary Tables 2 and 4 for more details on the strains included and the plasmid-harboring strains.

In total, from the 33 published datasets (Supplementary Table 1), 1,921 assembled genomes representing 14 abundant *L. monocytogenes* STs were included in this study. The ST with the lowest number of strains was ST204 (*n* = 52, Figure 1, Table 2, and Supplementary Table 2). The STs with the highest number of strains were ST9, ST5, and ST121 (*n* = 323, 286, 286, respectively). 45.7% (*n* = 877) of the strains in this study belonged to *L. monocytogenes* lineage I, and lineage II members comprised the remaining 54.3% (*n* = 1,044) of the strains. The strains originated from 32 countries across all six inhabited continents. Of these strains, 42% (*n* = 810) were collected in the United States, the most prevalent country of origin (Supplementary Figure 1), followed by Italy which accounted for 26% of the strains (*n* = 502) and Canada which accounted for 10% of the strains (*n* = 201). Seven countries were represented by only a single strain: Argentina (ST1), Brazil (ST204), Ecuador (ST6), Egypt (ST204), Finland (ST9), and Mexico (ST1). The strains in this study were isolated between 1958 and 2018 (Supplementary Figure 2). The number of strains isolated and sequenced per year was fairly consistent from 1986 to 2000 but experienced a marked increase from 2001 onward. The majority of strains were originally isolated from 2011 to 2015. All strains were sorted according to their source of isolation: clinical, environmental, food, or unknown origin. Clinical strains included human and animal listeriosis strains and represented 28% of the strains. Food-related strains comprised 37% of the total dataset, whereas 34% of the strains were environmental strains, originating from the natural environment and food production environments (FPEs; Figure 2A). The origin of 1% of the strains was not reported in the collected metadata.

**TABLE 2:** Number and percentage of potential plasmids identified in *Listeria monocytogenes* strains from different STs.

*Listeria monocytogenes* sequence type	*Listeria monocytogenes* lineage	Number of strains	Number of strains with putative plasmids	Percentage of strains with putative plasmids
121	II	286	263	92.0
5	I	286	254	88.8
8	II	67	53	79.1
3	I	58	45	77.6
204	II	52	37	71.2
9	II	323	159	49.2
6	I	147	66	44.9
7	II	108	46	42.6
120	II	62	25	40.3
155	II	74	29	39.2
14	II	72	25	34.7
2	I	123	19	15.4
1	I	209	15	7.2
4	I	54	1	1.9
Total		1,921	1,037	54.0

### Presence of Plasmids

Out of the 1,921 strains, 54.0% (*n* = 1,037) were identified as harboring potential plasmids (Table 2 and Supplementary Table 4). Broken down by lineage, 45.3% of the lineage I strains and 61% of the lineage II strains harbored plasmids. The ST with the highest percentage of strains bearing putative plasmids was ST121 at 92%. ST5, ST8, ST3, and ST204 also showed high rates of plasmid carriage with 88, 79, 78, and 71% of the strains carrying putative plasmids, respectively. In *L. monocytogenes* STs 14, 155, 120, 6, and 9, between 35 and 49% of the strains contained plasmids. A low (<15%) abundance of plasmids was found in the ST4, ST1, and ST2 strains in this study. The 1,037 identified putative plasmid-carrying strains were also analyzed according to the source of isolation. In contrast to the relatively even representation of *L. monocytogenes* strains from each of the three main isolation sources, the presence of plasmids in strains from different sources of origins was more unevenly distributed. Among all putative plasmids, 48% of the plasmids originated from environmental strains. Plasmids in food-related strains accounted for 39% of the putative plasmids, and plasmids from clinical strains made up 13% with the remaining 1% of plasmids originating from strains with an unknown source (Figure 2).

**FIGURE 3:**
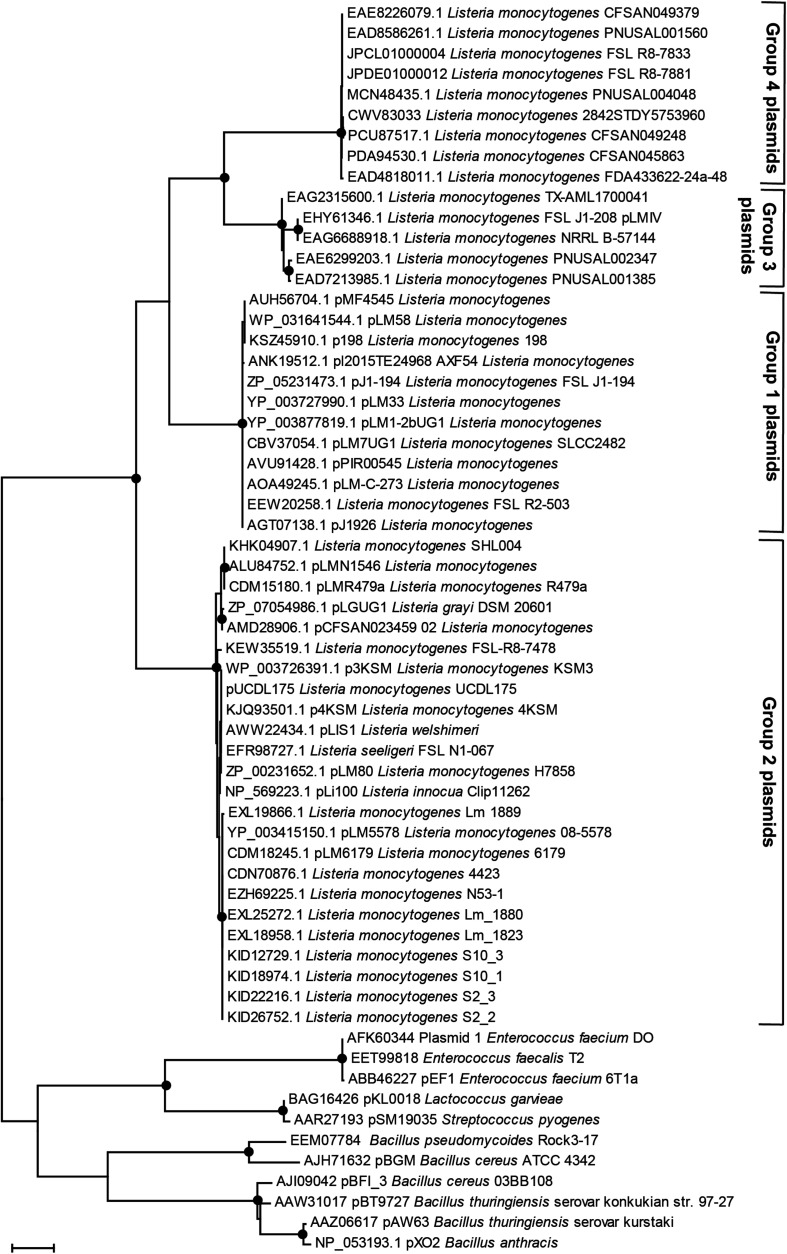
Phylogenetic relationships of plasmid replication protein RepA amino acid sequences. The evolutionary history was inferred by using the Maximum Likelihood method based on the JTT matrix-based model. The tree with the highest log-likelihood is shown. Initial tree(s) for the heuristic search were obtained automatically by applying Neighbor-Join and BioNJ algorithms to a matrix of pairwise distances estimated using a JTT model and then selecting the topology with superior log likelihood value. The tree is drawn to scale, with branch lengths measured in the number of substitutions per site. The analysis involved 61 amino acid sequences. All positions containing gaps and missing data were eliminated. Evolutionary analyses were conducted in MEGA7 (Kumar et al., 2016). The percentage of trees in which the associated taxa clustered together based on Maximum Parsimony and Maximum Likelihood is shown as black dots; the dots represent bootstrap values >90% using 1,000× resampling.

*Listeria* plasmids have been classified into two groups based on their replication protein RepA sequences (Kuenne et al., 2010). Out of the 1,037 strains harboring putative plasmids, 395 had a group 1 plasmid and 540 strains a group 2 plasmid (Supplementary Table 4). Based on their replication proteins, the group 1 plasmids are classified as Rep25 in the PLSDB database and the group 2 plasmids as Rep26. Within different STs, the prevalence of group 1 and group 2 RepA plasmid types was variable. Some STs contained mostly or exclusively group 1 (e.g., ST3, ST9, and ST1) or group 2 (e.g., ST8, ST120, ST121) RepA type plasmids, whereas other STs showed a more homogeneous distribution of RepA plasmid types. Interestingly, in a number of strains from different STs (*n* = 86), two *repA* genes were found in the plasmid contig(s) from the same strain (Supplementary Table 4). During the analyses of the RepA protein BLASTp searches, we identified additional RepA homologs that showed only between 55 and 62% amino acid identity to both group 1 and group 2 *Listeria* RepA sequences. We thus determined the phylogenetic relationship of plasmid RepA proteins to clarify the grouping of different RepA proteins. Phylogenetic analyses of RepA amino acid sequences from this study revealed consistent clustering of RepA proteins into four distinct, phylogenetically well-supported (based on maximum likelihood and maximum parsimony bootstrap values) groups (Figure 3). The four groups consisted of the established group 1 and 2 RepA plasmids, the newly identified group of putative RepA proteins, and a fourth group consisting of pLMIV-like RepA sequences. We thus suggest classifying the pLMIV-like plasmids as group 3 and the novel identified group of putative plasmids as group 4 RepA type plasmids.

Overall, putative group 4 plasmids were rare in our dataset and found in only eight strains (0.8% of all strains). Additional group 4 RepA proteins (with more than 89% amino acid identity) were present in 35 *L. monocytogenes* strains which were not part of our dataset. These strains were identified in NCBI GenBank as determined by BLASTp searches against NCBI GenBank nr (Figure 3, data not shown). A more detailed analysis of the group 4 RepA plasmid contigs in our dataset revealed that these RepA proteins were encoded on contigs that contained no annotated chromosomal genes, providing additional support that the group 4 plasmids actually represent true plasmids. Further emphasizing that these contigs are plasmids, the GC content ranged from 31.6 to 32.7%, which is lower than the average GC content of *L. monocytogenes* chromosomes (approximately 38.0%). Indeed, the majority of bacterial plasmids show a lower GC content compared to their chromosomal GC content (Shintani et al., 2015). In most cases, the putative group 4 plasmids were assembled into single contigs and seem to represent plasmids with a size of either approximately 55 or 69 kbp (Supplementary Figure 3). Most of the genes on these putative group 4 plasmids were uncharacterized. However, these contigs harbored a gene encoding a putative QacH QAC transporter showing 90% amino acid identity to QacH from Tn*6188* and 50% amino acid identity to BcrBC from pLM80. Interestingly, all putative group 4 plasmid contigs harbored several genes bearing approximately 40% amino acid identity to proteins encoded by genes found on the *Bacillus anthracis* plasmids pXO1 and pXO2. These included the type 4 secretion system components VirB2, VirD4, and VirB4, as well as other proteins from *Bacillus* plasmids.

### Size Range of *L. monocytogenes* Plasmids

**FIGURE 4:**
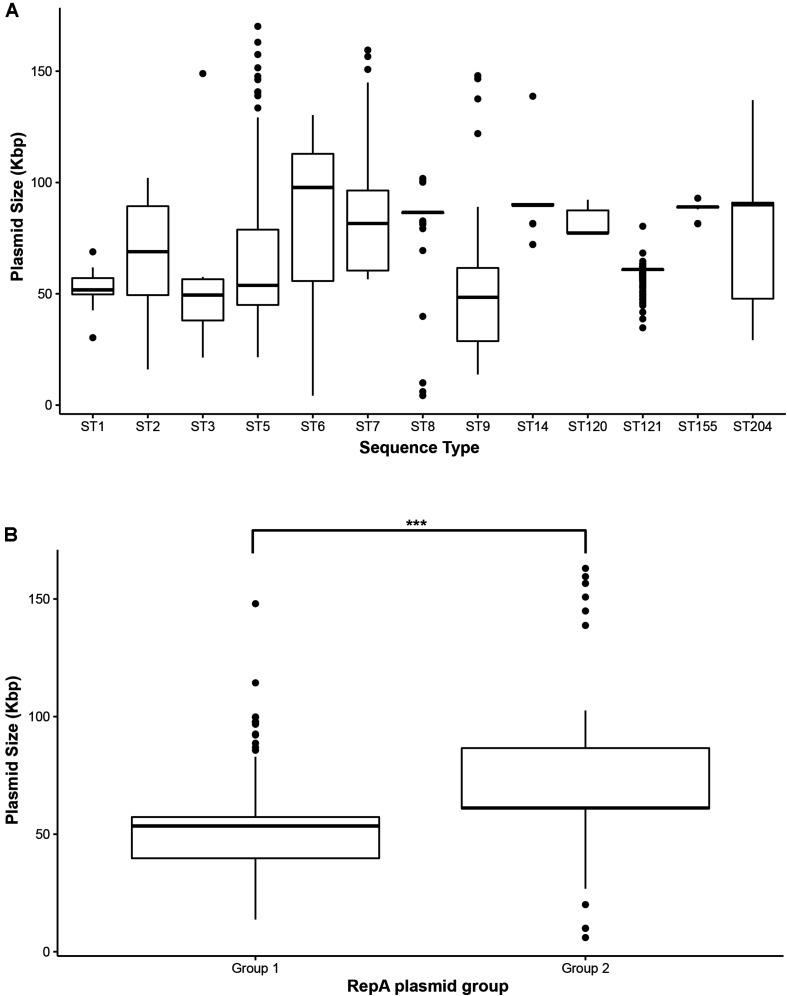
Size distribution of *Listeria monocytogenes* plasmids visualized as Box-Whisker plots. **(A)** Size distribution of plasmids per ST, **(B)** size distribution of group 1 and group 2 RepA plasmids. ***Indicates *p*-value < 0.001 (paired two-tailed Student’s *T*-test).

Overall, the predicted plasmid sizes based on the sizes of putative plasmid contigs ranged from approximately 4 kb (pLMST6-like) to 170 kb (Table 3 and Supplementary Table 4). The average and median sizes of all the plasmids were 65 and 61 kbp, respectively. Within different STs, the size ranges of the putative plasmids varied considerably. Some STs, such as ST2, ST5, ST6, ST8, and ST9, showed high variability in plasmids sizes, whereas other STs such as ST1, ST3, ST14, ST120, ST121, and ST155 showed lower variability (Figure 4). Group 1 plasmids had a median size of 53.5 kbp and were significantly (*p* < 0.001) smaller than the group 2 plasmids which had a median size of 61.1 kbp (Figure 4). The smallest characterized plasmids in this dataset were pLMST6-like plasmids with a size of 4.3 kb which were found in seven ST6 strains and in one ST8 strain. We did not identify homologs of other previously described small, antibiotic resistance-carrying plasmids in *L. monocytogenes* such as pIP823 or pDB2011 from *L. innocua* (Charpentier et al., 1999; Bertsch et al., 2013).

**TABLE 3:** Size metrics and RepA typing for the plasmids of different *Listeria monocytogenes* STs*.

	ST1	ST2	ST3	ST5	ST6	ST7	ST8	ST9	ST14	ST120	ST121	ST155	ST204
Plasmid size range (kbp)	30–69	16–102	21–149	21–170	4–130	56–159	4–101	13–148	72–138	76–92	34–80	81–93	29–137
Average plasmid size (kbp)	52.6	66.9	48.0	65.2	81.8	84.6	79.7	51.1	89.5	81.3	59.7	88.3	78.4
Median plasmid size (kbp)	54.0	68.9	49.4	53.8	97.7	81.6	86.6	48.4	89.8	77.3	60.9	89.0	90.0
Percentage of plasmids typed as group 1 RepA	73.3	26.3	97.8	55.5	42.4	26.1	0	88.7	8.0	0	1.9	0	16.2
Percentage of plasmids typed as group 2 RepA	6.7	36.8	0	24.8	7.6	73.9	98.1	9.4	92.0	100	98.1	100	75.7
Percentage of plasmids typed as group 1 and group 2 RepA	0	15.8	2.2	19.7	39.4	0	0	1.9	0	0	0	0	8.1
Percentage of plasmids typed as group 4 RepA	20.0	21.1	0	0	0	0	0	0	0	0	0	0	0

*As ST4 contained only one strain harboring a plasmid (group 4 RepA), it is not included in this table.

### Overall Conservation of Plasmids

Using BLASTn searches with various plasmids as query sequences, the conservation of plasmids within and between STs was determined. Not surprisingly, plasmid size and conservation varied highly within our dataset. However, we did still identify some highly conserved plasmids, including plasmid subtypes shared between certain strains within a given ST and derived from the same or similar source.

### Plasmids Shared Within STs

Fifty-four ST5 strains collected from the state of New York from 2005 to 2017 as part of a study analyzing *L. monocytogenes* strains in retail environments (Stasiewicz et al., 2015) harbored yet uncharacterized, putative 53 kbp plasmids. *L. monocytogenes* strains from ST8 have been shown to harbor plasmids in previous studies (Schmitz-Esser et al., 2015a; Fagerlund et al., 2016; Hingston et al., 2017, 2019). Here, we found virtually identical 86 kbp pLMR479a-like plasmids in 39 out of the 53 (73.6%) plasmid-carrying ST8 strains. These ST8 strains were derived from Italy, Denmark, Norway, France, Switzerland, Germany, Netherlands, Chile, and China and were isolated between 1996 and 2017. Plasmid pLM58, a recently identified plasmid found in an ST9 strain that confers increased heat tolerance (Pontinen et al., 2017), was present in seven ST9 strains from Canada, Ireland, Norway, and Chile isolated from 1991 to 2012 with >99.9% nucleotide identity and >99% coverage. ST9 strains are also known to harbor 25.5 kbp plasmids which possess more than 99.9% nucleotide identity and more than 99% coverage with pMF4545-like plasmids. These pMF4545-like plasmids were originally identified using long-read sequencing technology (Fagerlund et al., 2018). We identified these plasmids in 37 strains from Norway, Italy, and Switzerland collected from 2000 to 2014. We also found 16 ST120 strains collected from 1990 to 2011 from Canada and New Zealand that harbored a 77 kbp plasmid; this plasmid was highly similar to pLM5578 (Gilmour et al., 2010) with >99.8% nucleotide identity and >99% coverage. Finally, in agreement with previous reports, most *L. monocytogenes* ST121 strains harbored a highly similar, pLM6179-like 62 kbp plasmid. This plasmid was virtually identical (>99.9% nucleotide identity and >99% coverage) across 198 out of the 263 ST121 strains which harbored plasmids (75.3%). Plasmids were also conserved across time, as the plasmid pLM33 (Canchaya et al., 2010) was shared by seven ST3 strains from Italy, Switzerland, Spain, and Germany isolated over a time period spanning from 1966 to 2018. The pLM33 sequences were highly similar, with >99.9% nucleotide identity and >99% coverage.

### Plasmids Shared Between Different STs

In addition to identifying shared plasmids within an ST, we also aimed to identify plasmids found in different STs. The 50 kbp plasmids pLM7UG1 (*L. monocytogenes* SLCC2482, ST3) and pLM1-2cUG1 (*L. monocytogenes* SLCC2372, ST122) have previously been described to be highly similar and classified as pLisI (Kuenne et al., 2010). Here, we confirm those results and show that pLM7UG1 and pLM1-2cUG1 are virtually identical and are found in nine ST3 strains and four ST1 strains (1.4% of all strains harboring plasmids) (Supplementary Figure 4). The strains were derived from Italy, Germany, the United Kingdom, the United States, and Chile from 1966 to 2018. Notably, the pLM1-2cUG1-harboring strain SLCC2372 was isolated from a human in the United Kingdom in 1935 (Kuenne et al., 2013). Thus, these virtually identical pLisI-like plasmids have been circulating in *L. monocytogenes* for almost 90 years at least. Kuenne et al. (2010) also identified another cluster of four highly similar 57 kbp plasmids, termed pLisII. Here, using pLM1-2bUG1 as a query, we found pLisII-like plasmids (>99.9% nucleotide identity and >99% coverage) in 49 strains (4.7% of all strains harboring plasmids) from ST1, ST2, ST3, ST5, ST7, and ST9 (Supplementary Figure 5). The origins of these strains included the United States, Canada, Italy, Switzerland, Germany, China, and Chile, and the strains were isolated over a time period ranging from 1966 to 2018. Additional pLisII-like plasmids were also found in NCBI GenBank in other STs (Supplementary Figure 5). The pLisI-like plasmids (50 kbp) represent a subset of the larger pLisII plasmids (57 kbp); the pLisII plasmids harbor additional predicted stress-response genes such as a putative NADH peroxidase, a GbuC-like putative glycine-betaine transporter binding protein, and a putative heavy metal transporter which are absent from the pLisI plasmids (Kuenne et al., 2010) (Supplementary Figure 6).

Recently, the 81 kbp conjugative plasmid pLIS1 was described in *L. welshimeri*, and the authors noted the presence of a highly similar plasmid in *L. monocytogenes* strain CFSAN004330 (Korsak et al., 2019). It is important to mention that pLIS1 is also virtually identical to pLM80. Because the pLM80 sequence consists of two contigs, we utilized pLIS1 as a query sequence against the strains in our study. We identified pLIS1/pLM80-like plasmids (>99.9% nucleotide identity and >99% coverage) in 35 strains from ST5, ST7, ST9, ST14, and ST155 (Supplementary Figure 7) representing 3.4% of all strains harboring plasmids. The pLIS1/pLM80-like plasmids were identified in strains collected from 1990 to 2018 from the United States, Canada, and Italy. The BLASTn searches with pLIS1 as query indicated the presence of an additional, larger pLIS1/pLM80-like plasmid in other strains. The 90 kbp pLIS1 variants are characterized by the presence of additional genes, including a putative Clp-like protein, a putative NADH peroxidase, a putative riboswitch (see below), and a putative glycine-betaine transporter binding protein GbuC. This 90 kbp variant of pLIS1 was present in 85 strains (8.2% of all strains harboring plasmids) from ST2, ST5, ST6, ST7, ST9, ST14, ST155, and ST204 (>99.9% nucleotide identity and >99% coverage, Supplementary Figures 8, 9). The strains harboring the 90 kbp pLIS1 variant were isolated from the United States, Canada, France, Switzerland, Austria, Nigeria, Australia, and New Zealand between 1996 and 2018.

### Presence and Conservation of Plasmid Stress-Response Genes Across STs

For most of the *L. monocytogenes* plasmid genes, a putative function based on sequence similarity cannot be deduced. Based on previous publications, we selected nine *L. monocytogenes* plasmid genes or loci which have been demonstrated or suggested to be involved in various stress response mechanisms (Table 1). These loci of interest include genes encoding a putative multicopper oxidase (MCO) (Sitthisak et al., 2005), the cadmium efflux proteins CadA1 (Tn*5422*) and CadA2 (Lebrun et al., 1994; Parsons et al., 2018), the QAC resistance proteins BcrABC (Elhanafi et al., 2010), the heat shock protein ClpL (Pontinen et al., 2017), the triphenylmethane reductase Tmr (Dutta et al., 2014), a putative NADH peroxidase (NPR) (Hingston et al., 2019), and a putative riboswitch recently identified during transcriptome sequencing (Cortes et al., 2020). Overall, the presence of these different stress response genes on *L. monocytogenes* plasmids ranged from 35 to 60% on average but was highly variable ranging from 0 to 100% depending on the ST (Table 4). Interestingly, for all selected stress response genes, the nucleotide identity was 99% or greater. Most of the hits shared 99.9% or higher nucleotide identity, providing additional evidence for the high level of conservation of *Listeria* plasmid genes. Strikingly, 97% of the plasmids in this study contained either *cadA1* or *cadA2*. *gbuC*, *npr*, and the putative nickel and cobalt-binding (“NiCo”) riboswitch genes showed highly similar abundance patterns in the different STs (59–60% of all plasmids), raising the possibility these three genes are linked due to being in the same genetic module. In our study, *bcrABC* was found on average in 46% of the plasmids. All ST155 plasmids contained the *bcrABC* cassette, and a high percentage of plasmids carrying the *bcrABC* cassette (92%) was also observed in ST14 and ST204. In contrast, no ST8 or ST120 plasmid contained *bcrABC*, and *bcrABC* was found in only 1% of ST121 plasmids. On average, *clpL* was found on 40% of all plasmids in this dataset. 99% of ST121 plasmids possessed *clpL* as did all ST3 plasmids. Conversely, ST8 and ST155 plasmids did not carry the *clpL* gene. Finally, the presence of the *mco* gene was variable, with an average of 35% of all putative plasmids carrying this gene, ranging from 0% in ST155 plasmids to 96% in ST120 plasmids (Table 4). The arsenic resistance locus identified on the *L. innocua* plasmid pLI100 (Parsons et al., 2018) was not present on any of the plasmids in our dataset. BLASTn hits for this locus were found but were all present on chromosomal contigs. Similarly, we did not identify any homologs of the four internalin-like genes which are found on the *L. monocytogenes* plasmid pLMIV (den Bakker et al., 2012).

**TABLE 4:** Percentage of selected plasmid-encoded stress response genes by ST based on BLASTn.

ST	*cadA1* (%)	*cadA2* (%)	*tmr* (%)	*bcrABC* (%)	*clpL* (%)	*mco* (%)	*gbuC* (%)	*npr* (%)	Putative NiCo riboswitch (%)
1	77	8	8	8	77	77	38	38	38
2	21	58	53	53	16	5	37	37	37
3	100	2	4	4	100	62	44	44	42
5	37	69	31	79	30	20	61	61	61
6	2	88	71	76	2	2	39	41	39
7	41	59	63	63	22	41	43	43	43
8	94	0	0	0	0	89	87	91	83
9	90	14	23	32	70	33	63	65	62
14	8	92	92	92	8	8	80	80	80
120	100	0	0	0	76	96	96	96	96
121	99	1	1	1	99	2	2	2	2
155	0	100	100	100	0	0	93	93	93
204	16	81	92	92	16	16	86	86	86
Average	53	44	41	46	40	35	59	60	59

*All of the plasmid-encoded genes shown share more than 99% nucleotide identity to their query sequences. Percentages were calculated per number of strains harboring plasmids in each ST.*

**FIGURE 5:**
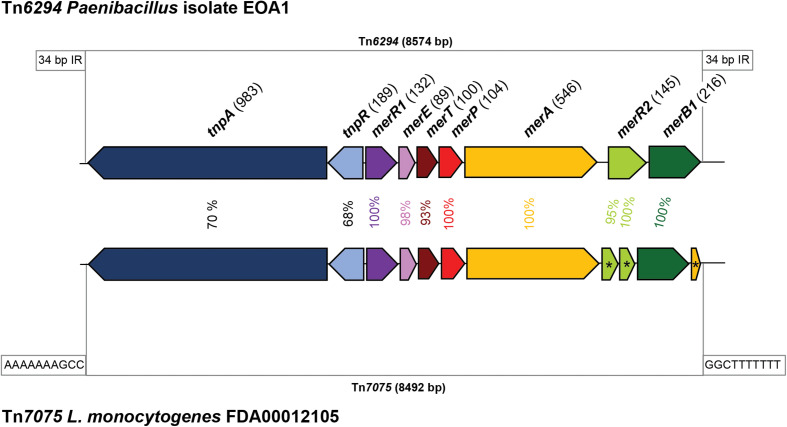
Genetic organization and similarity of mercury resistance transposons. The mercury resistance transposon Tn*6294* from the *Paenibacillus* strain EAO1 (Matsui et al., 2016) and the transposon Tn*7075* found on the putative plasmid in *Listeria monocytogenes* FDA00012105 are shown. Homologous genes are shown in the same color. Putative pseudogenes are indicated by an asterisk (*). The inverted repeats flanking the transposons are indicated.

In addition to identifying known and putative stress response, our dataset proved useful in identifying novel genetic features on *L. monocytogenes* plasmids. Through manual review of the annotations, we detected a putative mercury resistance locus on the plasmid of *L. monocytogenes* ST6 strain FDA00012105. This locus shows 98.7% nucleotide identity with the 8,574 bp mercury transposon Tn*6294* from the unclassified *Paenibacillus* strain EAO1, sharing 4,700 bp of overlap with the mercury resistance genes (Matsui et al., 2016) (Figure 5). The amino acid identities between Tn*6294* and the *L. monocytogenes* homolog ranged from 68 to 70% for the transposases TnpA and TnpB and was even higher for the mercury resistance proteins, ranging from 93 to 100%. In *L. monocytogenes*, the locus consists of 8,492 bp and includes the following proteins: the regulatory protein MerR1, the MerETP mercury transporters, the mercury reductase MerA, and the organomercurial lyase MerB1. However, the transcriptional regulator MerR2 was predicted to be a pseudogene, and an additional pseudogene copy of *merA* was also present in the transposon. Interestingly, these two genetic elements were predicted to be pseudogenes in all *L. monocytogenes* Tn*6294*-like copies in our dataset. Based on the differences between the *L. monocytogenes* homologs and Tn*6294*, the *L. monocytogenes* mercury resistance transposon was classified as Tn*7075* (Tansirichaiya et al., 2019). Similar to Tn*6294*, Tn*7075* is flanked by inverted repeats, which are, however, shorter (10 bp) than the inverted repeats in Tn*6294* (34 bp). Tn*7075* was present in 26 plasmids in our dataset (2.5% of all plasmids).

## Discussion

Here, we present a large and representative dataset consisting of 1,921 *L. monocytogenes* genomes, including strains of 14 STs collected from 32 countries over a period of 60 years. Thus, this is by far the most extensive dataset used for a plasmid survey in *L. monocytogenes*. Our dataset also contains a roughly equal representation of strains from all three characterized isolation sources, and each ST was represented by a large number of strains (52 at minimum with an average of 137). Previous molecular studies examining the presence of plasmids in *Listeria* were limited in scope being based on significantly smaller datasets or focusing either on specific STs or specific conditions. Research conducted by Hingston et al. (2017) examined 166 *L. monocytogenes* genome sequences from primarily food and FPE strains. Chen et al. (2020) analyzed the genomes of 201 *L. monocytogenes* strains isolated from RTE foods for the presence of plasmids. Furthermore, until now, only two studies have performed surveys of plasmids in the genus *Listeria*. However, these studies exclusively analyzed plasmid-carrying strains and had far smaller datasets. Kuenne et al. (2010) analyzed 14 *Listeria* plasmids, and more recently, Hingston et al. (2019) analyzed 93 *L. monocytogenes* strains with plasmids.

While this study represents a major contribution to our understanding of plasmids in *L. monocytogenes*, it should be noted that there remain some limitations to the scope of our dataset and to our sequence analysis approach. First, it should be highlighted that our dataset consists only of previously assembled genomes. While many submissions contained raw *L. monocytogenes* reads, assembling genomes from these reads would have drastically increased the bioinformatic effort. Next, there remain possible biases regarding the representative nature of this dataset. For example, we acknowledge that by the inclusion of all genomes from outbreak investigations (which would theoretically be highly similar) we may have introduced a potential bias in the frequency of plasmid carriage. However, plasmid carriage can vary even between strains isolated from the same outbreak, as evidenced in the 2008 Canadian deli meat outbreak where human, food, and environmental outbreak strains differed regarding the presence of the plasmid pLM5578 (Gilmour et al., 2010). Moreover, excluding outbreak strains altogether would introduce an additional bias, particularly for the hypervirulent STs that are by nature typically collected and sequenced as part of investigating disease outbreaks. Regardless, as detailed below, our results for plasmid presence are similar to what other studies (using smaller datasets) have reported and thus, provide support that our dataset is representative.

More generally, datasets like ours are dependent on the availability of metadata associated with sequenced genomes. Additionally, it is possible that plasmids or plasmid genes that are more distantly related to our query sequences of known *Listeria* plasmids might not be detected in a BLASTn analysis. This is because the requirements for nucleotide BLAST comparisons are inherently more stringent, meaning that a match must possess a highly similar sequence matching the query. We mitigated this potential limitation by using a diverse pool of plasmids from different STs as our query sequences; in this way, plasmids could be identified if they were either from or partially related to plasmids from a different ST (Table 2). Furthermore, a number of previous reports have demonstrated that *L. monocytogenes* plasmids are often highly conserved; *L. monocytogenes* plasmids also often possess a modular structure in which modules themselves are highly conserved and shared between different plasmids (Kuenne et al., 2010; Fox et al., 2016; Hingston et al., 2017, 2019; Rychli et al., 2017; Palma et al., 2020). Indeed, our results show that the shared plasmid modules and genes show at least 99% nucleotide identity. Therefore, BLASTn can reliably identify plasmid contigs in *L. monocytogenes* due to the parts of the plasmid which are shared between the query and the subject. Additionally, BLASTp searches using the plasmid replication protein RepA as a query provided an additional approach to identify plasmid contigs since homologs with lower similarity can be detected by protein BLAST (compared to nucleotide BLAST). Indeed, the BLASTp searches allowed us to identify the putative group 4 subgroup of plasmids.

The strains selected for this study were derived from 14 *L. monocytogenes* STs which were found to be abundant based on recent large-scale studies analyzing the abundance of *L. monocytogenes* (Henri et al., 2016; Moura et al., 2016; Bergholz et al., 2018; Painset et al., 2019). By including strains from these abundant and diverse STs, *L. monocytogenes* diversity is adequately represented in our dataset. Nevertheless, in the future, the analysis of additional STs (such as ST321, ST37, and ST101) would expand our current database and analyses. Another limitation of our plasmid identification approach is that the analyses were conducted *in silico* only. All analyses conducted were sequence-based, and the actual strains in the dataset are not available in our laboratory. Thus, we cannot and have not been able to verify experimentally that these strains do in fact contain plasmids. Therefore, any unverified plasmids in our dataset should be considered as putative plasmids. Moreover, the majority of the genomes included in this analysis are draft genomes and have not been closed, often resulting in chromosomal and plasmid assemblies spread across multiple contigs. This makes plasmid identification in strains with draft genomes difficult and raises the question of whether contigs are truly part of a plasmid or are instead segments of plasmids that have been incorporated into the chromosome. Incorporation of plasmid genes and parts of plasmids into the chromosome has been documented previously in *L. monocytogenes* (Rychli et al., 2014). However, in this study, we observed very few cases of putative plasmid integration into chromosomes. Therefore, in spite of the limitations mentioned above, the combination of multiple BLAST searches, annotation and analysis of representative plasmid subtypes, and the previously reported high conservation and modular structure of *L. monocytogenes* plasmids together suggest that our methodology reliably identified putative plasmid contigs. In addition, our dataset will likely be useful for future analysis, including the identification of genes of interest. Automated annotations can be inaccurate and require manual verification of genes of interest. For analysis of known target genes of interest speculated to be present on *L. monocytogenes* plasmids (or chromosomes), this is a large and comprehensive data set which can be utilized using both BLASTn and BLASTp analyses allowing for high throughput searches.

Overall, 54.0% (*n* = 1,037) of the 1,921 strains were found to harbor potential plasmids (Table 2). The abundance of plasmids in our study was similar to what has been previously reported for *L. monocytogenes*. Hingston et al. (2017) found that 55% of 166 strains from various *L. monocytogenes* STs contained a plasmid, and similarly, 48% of 201 *L. monocytogenes* RTE strains were found to harbor plasmids (Chen et al., 2020). A higher plasmid prevalence was observed in persistent *L. monocytogenes* strains from French seafood processing plants, with plasmids present in 70% of 94 strains (Palma et al., 2020). Similarly, all 42 strains from a study investigating the evolution of *L. monocytogenes* in a salmon processing facility harbored plasmids (Harrand et al., 2020). Non-sequencing-based surveys reported plasmids in 28–78% of the analyzed strains (Kolstad et al., 1991, 1992; Lebrun et al., 1992; McLauchlin et al., 1997; Harvey and Gilmour, 2001). In agreement with the literature, the percentage of plasmid-carrying strains was significantly higher in environmental and food strains compared to *L. monocytogenes* strains collected from clinical settings (Kolstad et al., 1992; Lebrun et al., 1992). We also show that plasmids are more abundant in lineage II than in lineage I strains, confirming previous reports (Hingston et al., 2017, 2019; Chen et al., 2020). Thus, the results from this study and those from previous studies show that plasmids are common in *L. monocytogenes* and can be particularly abundant in food and FPE strains.

Previously, small (<10 kbp) plasmids bearing genes involved in stress survival have been identified in *L. monocytogenes.* For example, the plasmids pIP823 and pDB2011 encode antibiotic resistance genes (Charpentier et al., 1999; Bertsch et al., 2013), and the benzalkonium chloride resistance locus *bcrABC* is encoded on pLMST6 (Kremer et al., 2017). We found no homologs of the small antibiotic resistance carrying plasmids in our dataset, indicating that those small plasmids are rare in *L. monocytogenes*. This phenomenon has also been observed before in a smaller dataset (Hingston et al., 2019). However, we did find virtually identical pLMST6-like plasmids in seven ST6 and in one ST8 strain (0.7% of all strains harboring plasmids). Corroborating this finding, a low abundance (1.6% of 439 strains) of pLMST6 has also been reported recently (Kropac et al., 2019).

Analyzing the prevalence of plasmids within *L. monocytogenes* STs revealed that the highest rate of plasmid carriage occurred in ST121 with 92% of the 287 strains carrying possible plasmids. This agrees with previous reports. One prior study found that 81.4% of 70 analyzed ST121 strains carried a plasmid (Rychli et al., 2017), and another study identified plasmids in 76% of the ST121 strains analyzed (Palma et al., 2020). In our study, ST5, ST8, ST3, and ST204 also showed high abundances of potential plasmids with prevalences of 88, 79, 78, and 71% in each strain, respectively. Similar results were reported by Hingston et al. (2017) where plasmids were found in more than 80% of ST3 and ST5 strains and in 64% of ST8 strains. Similarly, Chen et al. (2020) found that 100% of the ST5 strains they analyzed harbored a plasmid, and a high abundance of potential plasmids in ST8 strains was reported earlier (Fagerlund et al., 2016; Hingston et al., 2017). While the study by Chen et al. (2020) included only ten ST5 strains, our study included 287 ST5 strains. The results obtained here for ST204 are similar to what has been indicated by Fox et al. (2016) who found that 86.7% of the ST204 strains studied carried plasmids. Similarly, plasmids were discovered in 92% of the ST204 strains analyzed in a recent study (Palma et al., 2020). Again, it should be noted that the study by Fox et al. (2016) only examined 15 ST204 strains and that the study by Palma et al. (2020) had 22 ST204 strains. In contrast, our dataset for ST204 is based on 52 strains. In *L. monocytogenes* STs 14, 155, 120, 6, and 9 between 35 and 50% of the strains contained plasmids. A higher abundance of plasmids in ST155 strains (69% compared to 39% in our study) was reported by Chen et al. (2020). A low (<15%) abundance of plasmids was found in the ST4, ST1, and ST2 strains in this study. In line with this, Hingston et al. (2017) found no plasmids in their ST1, ST2, and ST4 strains, and Chen et al. (2020) also discovered a low abundance of plasmids in ST1 strains. Thus, our study strengthens the conclusions drawn in previous studies regarding the plasmid prevalence within individual *L. monocytogenes* STs.

Previously, *Listeria* plasmids have been classified into two distinct groups based on their RepA amino acid sequences (Kuenne et al., 2010). Here, we provide preliminary evidence for the presence of two additional groups of *Listeria* plasmids based on RepA protein phylogeny and genome sequencing data. The first of these novel putative RepA plasmid types is group 3. We propose pLMIV as a representative and characterized plasmid of this group (den Bakker et al., 2012). Group 4 consists of a group of novel, yet uncharacterized, putative *L. monocytogenes* plasmids with some elements homologous to elements found in *B. anthracis* plasmids. However, the existence of the putative group 4 plasmids needs to be verified in future studies as the experimental verification of these plasmids was beyond the focus of the current study. Based on our results, these novel group 3 and group 4 plasmids are less abundant compared to group 1 and 2 plasmids.

Our results suggest that most plasmid-carrying *L. monocytogenes* strains harbor one plasmid. However, we found several strains (*n* = 86) in this study that contained two *repA* genes, mostly on separate putative plasmid contigs which may indicate the presence of two plasmids within a single isolate. The existence of single strains harboring two plasmids has been reported previously for some *L. monocytogenes* strains (Kolstad et al., 1991; Lebrun et al., 1992; Romanova et al., 2002; Hingston et al., 2017; Korsak et al., 2019; Harrand et al., 2020). Alternatively, it is possible that the strains identified here with two RepA-encoding genes may harbor a single composite plasmid which possesses two different *repA* genes instead of two distinct plasmids; this phenomenon has been documented in the plasmid from *L. monocytogenes* N1-011A which was sequenced using Pacific Biosciences long-read sequencing technology (Korsak et al., 2019). Again, it is important to note that the potential presence of two plasmids in a single strain has not been experimentally confirmed in this study. Based on the references above and what conclusions we can draw from our results, the occurrence of strains with more than a single *repA* gene appears to be relatively uncommon. Therefore, further research into the possibility of the presence of multiple plasmids or composite plasmids within a single *L. monocytogene*s strain is necessary.

The conservation of plasmids was highly variable. Previous studies have shown a modular structure of *Listeria* plasmids with high conservation of the different modules that are shared between different plasmids (Kuenne et al., 2010; Fox et al., 2016; Hingston et al., 2019). Some earlier studies have shown that plasmids within a ST can be highly similar and, in some cases, virtually identical (Fagerlund et al., 2016; Fox et al., 2016; Hingston et al., 2017, 2019; Rychli et al., 2017; Palma et al., 2020). Here, we provide evidence for the presence of highly conserved plasmids within certain STs. For example, 62 kbp plasmids were found in unrelated ST121 strains, and these were virtually identical to pLM6719. This phenomenon was also reported recently for ST121 strains not included in our study (Palma et al., 2020). Similarly, we also observed 86 kbp plasmids which were highly similar to pLMR479a in various unrelated ST8 strains. Additionally, other described *L. monocytogenes* plasmids were found in unrelated strains within a specific ST. Such plasmids included pLM58 and pMF45445, which are both found in ST9 strains, and pLM33, which is found in ST3 strains.

In addition to identifying highly conserved plasmids present within certain STs, we also identified virtually identical plasmids that were shared between strains from different *L. monocytogenes* STs derived from different years and different countries. These plasmids included the 50 kbp pLM7UG1-like plasmids and the closely related 57 kbp pLM1-2bUG1 plasmids, which were found in 1.4 and 4.7% of all strains harboring plasmids, respectively. pLM1-2bUG1-like plasmids, designated as pLMG1-7, were also found to be the most abundant plasmid subtype and were present in strains from multiple STs, countries, and years of isolation (Hingston et al., 2019). Recently, the 81 kbp conjugative plasmid pLIS1 was described in *L. welshimeri* (Korsak et al., 2019); pLIS1 is virtually identical to pLM80 (Nelson et al., 2004). Here, we show the presence of pLIS1/pLM80-like plasmids in 3.4% of all strains harboring plasmids. We also identified a 90 kbp pLIS1/pLM80 variant which we found to be present in 8.2% of all strains harboring plasmids. Indeed, this 90 kbp pLIS1/pLM80 plasmid was recently found to be prevalent in *L. monocytogenes* ST155 and ST204 strains from seafood processing plants in France (Palma et al., 2020). It should be noted that the strains in the study by Palma et al. (2020) were not part of our dataset. Thus, based on the high abundances of the 90 kbp pLIS1/pLM80 plasmids in a number of independent studies, an important contribution of these plasmids to stress survival may be likely. Although the 90 kbp pLIS1/pLM80 plasmids have not yet been functionally characterized, the size and presence of these plasmids were verified using long-read sequencing technology in *L. monocytogenes* strain OB080183, an ST6 food isolate (Kwon et al., 2020). OB080183 was not part of our dataset but was included in the plasmid alignment shown in Supplementary Figure 8. Thus, our results demonstrate that certain nearly identical plasmids are shared within the genus *Listeria* and have been circulating worldwide over the past few decades. The broad distribution of these highly conserved plasmids in food and FPE strains suggests that these plasmids may be particularly beneficial for the survival of *L. monocytogenes* in food and FPEs.

Until now, little information existed regarding the function of *Listeria* plasmids in general or of specific plasmid-encoded genes. We thus analyzed our dataset for the presence of known or candidate stress response genes using BLASTn. Previously, the study of cadmium detoxification by *Listeria* plasmids and the role of cadmium ATPases in this process have resulted in the identification of the transposon Tn*5422* (Lebrun et al., 1994; Parsons et al., 2020). Overall, the *cadA1* and *cadA2* genes were present in 97% of all plasmids in our dataset, making them the most abundant stress response gene identified. These results corroborate previous research where plasmid-derived cadmium resistance was experimentally confirmed in 95.3% of the *L. monocytogenes* strains studied (Lebrun et al., 1992). Likewise, Hingston et al. (2019) found the *cadA1* and *cadA2* genes to be highly prevalent on *L. monocytogenes* plasmids, and other studies have also found plasmids to be highly correlated with cadmium resistance (McLauchlin et al., 1997; Harvey and Gilmour, 2001; Mullapudi et al., 2010). Interestingly, a high prevalence of cadmium resistance genes has been observed in many *L. monocytogenes* strains originating from food and FPEs (Pirone-Davies et al., 2018; Parsons et al., 2020). The above observations of plasmid-derived cadmium resistance and prevalence of cadmium resistance in food strains has led to the suggestion of an association between cadmium resistance genes and the persistence of *L. monocytogenes* (Pirone-Davies et al., 2018; Parsons et al., 2020); however, a potential function for cadmium resistance genes in *L. monocytogenes* persistence has yet to be elucidated. Thus, the contribution of cadmium resistance genes to the survival of *L. monocytogenes* in food and FPEs, both of which are habitats with negligible levels of exposure to cadmium, will need to be investigated in future studies.

The next most abundant stress response genes on *L. monocytogenes* plasmids encoded the putative glycine-betaine transporter binding protein GbuC, the putative NADH peroxidase Npr, and a putative, novel, uncharacterized NiCo riboswitch. All three genes showed highly similar abundance patterns and were found in 59–60% of all plasmids. The similar frequencies of these three genes suggest that they are part of one stress response module or are otherwise linked. Although all three genes are functionally uncharacterized, a putative role of their products in osmotolerance (GbuC) and oxidative stress (NADH peroxidase and the putative riboswitch) is likely. Indeed, *gbuC* (also referred to as *proW*) was significantly upregulated in response to salt stress, and *npr* was shown to be upregulated under both salt and acid stress (Hingston et al., 2019). Transcriptome sequencing in our lab examining the gene expression of *L. monocytogenes* strain R479a harboring pLMR479a showed that the putative NiCo riboswitch was significantly upregulated in response to lactic acid stress (pH 3.4) (Cortes et al., 2020).

*Listeria monocytogenes* is also often exposed to disinfectants such as QACs which are often used in food production or retail facilities as well as in household products. Therefore, increased tolerance to QACs and other disinfectants can be beneficial or required for *L. monocytogenes* strains to survive in food production facilities (Martinez-Suarez et al., 2016; Moretro et al., 2017; Maury et al., 2019). Previously, the *bcrABC* cassette was identified and functionally characterized on pLM80 (Elhanafi et al., 2010) and on other *L. monocytogenes* plasmids (Xu et al., 2016; Minarovicova et al., 2018; Korsak et al., 2019). Here, we found *bcrABC* on 46% of all plasmids. Interestingly, *bcrABC* was particularly abundant in ST14, ST155, and ST204 strains. A recent study found *bcrABC* on 41.5% of all strains, and many instances of the *bcrABC* cassette were plasmid-encoded (Cooper et al., 2020). Similar to the results obtained in our current study, these authors also found *bcrABC* to be particularly abundant in ST155 and ST204 strains.

We also searched our dataset for homologs of *clpL*, which encodes a heat shock protein, and *mco*, which encodes a MCO. ClpL is encoded on the *L. monocytogenes* plasmid pLM58 and has been demonstrated to confer increased heat stress tolerance (Pontinen et al., 2017). We found *clpL* homologs on 40% of all plasmids with a high abundance (>99%) in ST3 and ST121 plasmids. Additionally, the *clpL* gene on the *L. monocytogenes* plasmid pLM6179 was significantly upregulated in response to lactic acid stress (pH 3.4) (Cortes et al., 2020). Similarly, Hingston et al. (2019) observed upregulation of *clpL* in response to NaCl and low pH. These data suggest that the *L. monocytogenes* plasmid-encoded ClpL proteins may be involved in resistance to more stressors than just heat, although this hypothesis will need to be verified in future studies. We identified genes encoding MCOs in 35% of all plasmids in our study. The MCOs identified on *L. monocytogenes* plasmids share 95% amino acid identity with a homolog of the *Staphylococcus aureus* strain ATCC12600 MCO which is involved in both copper resistance and oxidative stress response (Sitthisak et al., 2005). Additionally, an identical putative *mco* was upregulated when exposed to mild (pH 5) (Hingston et al., 2019) and under more severe (pH 3.4) acid stress (Cortes et al., 2020). Similar to ClpL, these data indicate that MCOs may have a more diverse, yet unknown function in stress response which extends further than their canonical functions. Future research into the function of the *L. monocytogenes* plasmid MCOs is thus warranted.

Finally, we identified the presence of the Tn*7075* putative mercury resistance transposon on 2.5% of all *L. monocytogenes* plasmids. So far, increased tolerance of *L. monocytogenes* strains to mercury has been described in only one study (Lebrun et al., 1992); another study recently mentioned the presence of a putative mercury resistance locus in *L. monocytogenes* plasmids but did not provide any additional details (Hingston et al., 2019). Thus, until now, no data have been published regarding the molecular mechanisms of mercury resistance in *L. monocytogenes*. Based on the high similarity of the mercury resistance proteins to those encoded by the experimentally characterized Tn*6294* (93–100% amino acid identity), it is reasonable to assume that the *L. monocytogenes* Tn*7075* is similarly responsible for increased mercury tolerance. Interestingly, the merR2 proteins in all Tn*7075* copies identified here were predicted pseudogenes, potentially indicating that the regulatory function of merR2 is not required in *L. monocytogenes*. The additional, partial (42 amino acids) MerA copy encoded by the *L. monocytogenes* Tn*7075* shows 92% amino acid identity to the C-terminal segment of the full-length MerA (546 amino acids). This partial MerA copy is present in all *L. monocytogenes* Tn*7075* and may be the result of recombination events during integration. Previous research has demonstrated that Tn*6294* and other related mercury resistance transposons are widespread in many different species of the *Bacillales* order (Matsui et al., 2016). We thus speculate that Tn*7075* has been transferred horizontally into the genus *Listeria* as the GC content of Tn*7075* (excluding the region of the transposases) is 41.4%; this value is a significantly higher than the average GC content of *L. monocytogenes* plasmids and chromosomes. A function of this locus in mercury resistance will need to be verified in future studies.

## Conclusion

Here, we present an in-depth, comparative, *in silico* analysis of plasmids based on 1,921 *L. monocytogenes* genomes derived from a variety of STs, countries, years, and isolation sources. We determined that an average of 54% of the *L. monocytogenes* strains in this dataset harbored plasmids. This value ranged from 1 to 92% of the strains depending on ST, indicating that some *L. monocytogenes* STs are significantly more likely to carry plasmids than other STs. Overall, plasmids were significantly more abundant in *L. monocytogenes* strains from food, FPEs, and environmental sources compared to clinical strains. *L. monocytogenes* plasmids harbor a variety of stress response genes which were highly conserved across STs and different plasmids, but these genes were not uniformly or consistently found among STs. Our results thus confirm a high degree of modularity in *L. monocytogenes* plasmids. We show that certain *L. monocytogenes* plasmids are shared and highly conserved between *L. monocytogenes* strains on a large scale and across STs, suggesting that these plasmids provide important advantages for *L. monocytogenes* survival in food and FPEs.

## Data Availability Statement

The original contributions presented in the study are included in the article/Supplementary Material, further inquiries can be directed to the corresponding author.

## Author Contributions

SS-E designed and conceived the experiments. BC, JA, and SS-E analyzed the data and wrote the manuscript. All authors contributed to the article and approved the submitted version.

## Conflict of Interest

The authors declare that the research was conducted in the absence of any commercial or financial relationships that could be construed as a potential conflict of interest.
