# Supplementary material for: A Large-Scale Sequencing-Based Survey of Plasmids in Listeria monocytogenes Reveals Global Dissemination of Plasmids
Source: Front Microbiol. 2021 Mar 12;12:653155. doi: 10.3389/fmicb.2021.653155 (PMC7994336; doi:10.3389/fmicb.2021.653155)
Supplement: Supplementary file 2 [file Data_Sheet_2.PDF]

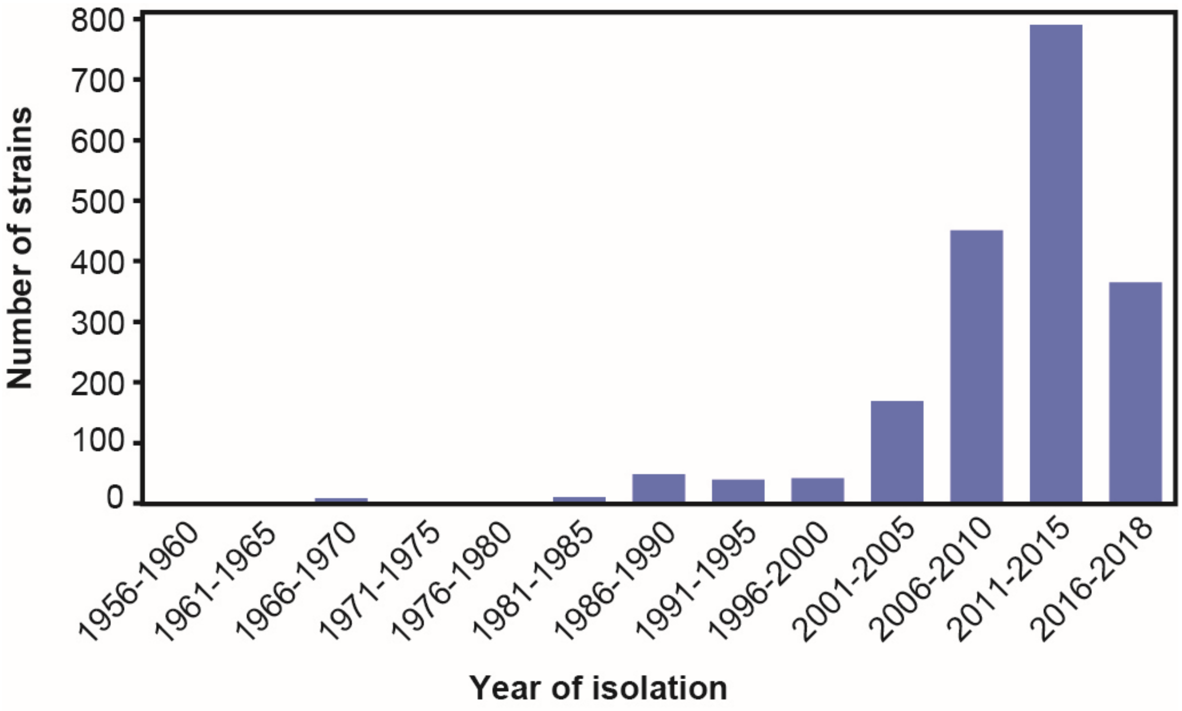

Supplementary Figure 2. Year of origin of the *L. monocytogenes* strains included in this study, grouped in five-year increments.
