# Supplementary material for: A Large-Scale Sequencing-Based Survey of Plasmids in Listeria monocytogenes Reveals Global Dissemination of Plasmids
Source: Front Microbiol. 2021 Mar 12;12:653155. doi: 10.3389/fmicb.2021.653155 (PMC7994336; doi:10.3389/fmicb.2021.653155)
Supplement: Supplementary file 4 [file Data_Sheet_4.PDF]

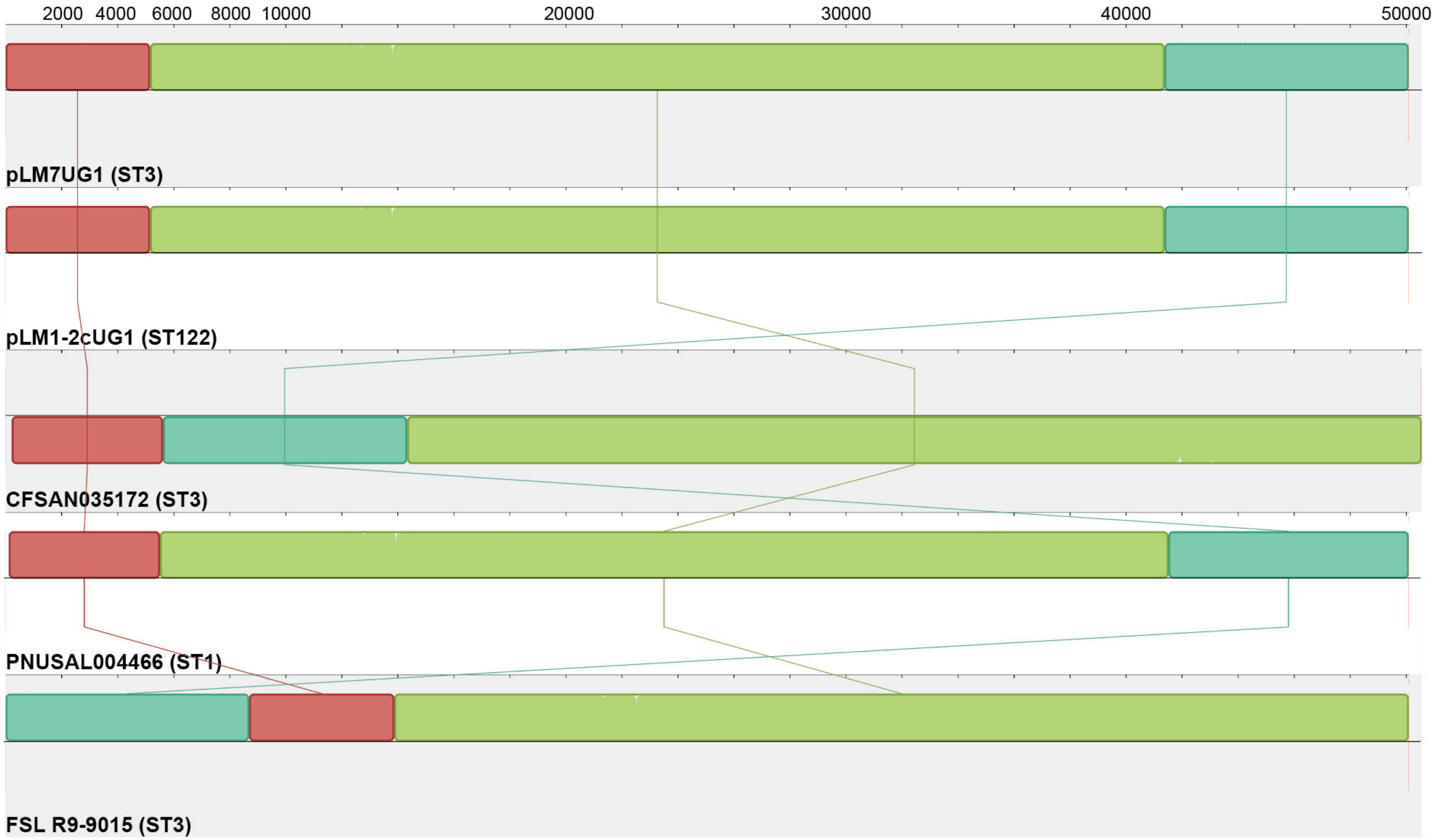

Supplementary Figure 4. Alignment of pLM7UG1 and related pLisI plasmids. STs are indicated in parentheses. The plasmids were aligned with MAUVE (Darling et al, 2010, PLoS One;5(6):e11147.). Homologous regions have the same color and the height of the blocks correlates with the conservation level of the regions for each plasmid. The size in base pairs is indicated at the top of the alignment.
