# Supplementary material for: A Large-Scale Sequencing-Based Survey of Plasmids in Listeria monocytogenes Reveals Global Dissemination of Plasmids
Source: Front Microbiol. 2021 Mar 12;12:653155. doi: 10.3389/fmicb.2021.653155 (PMC7994336; doi:10.3389/fmicb.2021.653155)
Supplement: Supplementary file 10 [file Data_Sheet_10.PDF]

Supplementary Table 1. Publications used for selection of *L. monocytogenes* isolates included in this study. Isolation sources: F: Food, C: Clinical, E: Environmental, U: Unknown. The detailed references can be found below the Table.

Reference	Title	Country/countries	Isolate year range	Isolation source
Allam et al. (2018)	Whole Genome Sequences of *Listeria monocytogenes* Sequence Type 6 Isolates Associated with a Large Foodborne Outbreak in South Africa, 2017-2018	South Africa	2017-2018	FCE
Bergholz et al. (2018)	Determination of Evolutionary Relationships of Outbreak-Associated *Listeria monocytogenes* Strains of Serotypes 1/2a and 1/2b by Whole-Genome Sequencing	USA, Switzerland, Italy, Canada, Finland, Denmark	1966-2012	FCE
Burall et al. (2017)	A clade of *Listeria monocytogenes* serotype 4b variant strains linked to recent listeriosis outbreaks associated with produce from a defined geographic region in the US	USA, Australia, Canada	2010-2015	FCE
Chen et al. (2017)	Comparative Genomics Reveals the Diversity in Restriction-Modification Systems and DNA Methylation Sites in *Listeria monocytogenes*	USA	1989-2011	FCE
Chiara et al. (2014)	Draft Genome Sequences of Six *Listeria monocytogenes* Strains Isolated from Dairy Products from a Processing Plant in Southern Italy	Italy	2012	F
Fagerlund et al. (2016)	Genome Analysis of *Listeria monocytogenes* Sequence Type 8 Strains Persisting in Salmon and Poultry Processing Environments and Comparison with Related Strains	Norway, Denmark, China, Italy, Canada, Switzerland	1994-2014	FE
Fox et al. (2016)	Comparative Genomics of the *Listeria monocytogenes* ST204 Subgroup	Australia, Ireland	2000-2015	FCE
Halbedel et al. (2018)	Whole-Genome Sequencing of Recent *Listeria monocytogenes* Isolates from Germany Reveals Population Structure and Disease Clusters	Germany	2007-2017	C
Hilliard et al. (2018)	Genomic Characterization of *Listeria monocytogenes* Isolates Associated with Clinical Listeriosis and the Food Production Environment in Ireland	Ireland	2013-2015	CE
Hingston et al. (2017)	Genotypes Associated with *Listeria monocytogenes* Isolates Displaying Impaired or Enhanced Tolerances to Cold, Salt, Acid or Desiccation Stress	Canada, Switzerland	1990-2013	FCE
Horlbog et al. (2018)	Whole-Genome Sequences of Six *Listeria monocytogenes* Strains Isolated from Food	Switzerland	2011-2014	F
Hyden et al. (2016)	Whole genome sequence-based serogrouping of *Listeria monocytogenes* isolates	Austria, France, Denmark, USA, Germany, Canada, UK, New Zealand	1958-2015	FCE
Kim et al. (2018)	Genetic diversity and virulence profiles of *Listeria monocytogenes* recovered from bulk tank milk, milk filters, and milking equipment from dairies in the United States (2002-2014)	USA	2002-2014	FE
Kwong et al. (2016)	Prospective Whole-Genome Sequencing Enhances National Surveillance of *Listeria monocytogenes*	Australia	1995-2015	FCE
Lomonaco et al. (2015)	The evolution and epidemiology of *Listeria monocytogenes* in Europe and the United States	Italy	2002-2015	FCE
Lopez-Alonso et al. (2019)	Whole-Genome Sequencing of Seven *Listeria monocytogenes* Strains from Different Stages of a Poultry Meat Production Chain	Spain	2004	E
Maury et al. (2016)	Uncovering *Listeria monocytogenes* hypervirulence by harnessing its biodiversity	France, Italy, USA	1997-2012	FCE
Morganti et al. (2016)	Processing-Dependent and Clonal Contamination Patterns of *Listeria monocytogenes* in the Cured Ham Food Chain Revealed by Genetic Analysis	Italy	2011-2014	E

Moura et al. (2017)	Whole genome-based population biology and epidemiological surveillance of *Listeria monocytogenes*	Canada, Denmark, Finland, France, Italy, Switzerland, UK, USA	1960-2015	FCE
Muhterem-Uyar et al. (2018)	New Aspects on *Listeria monocytogenes* ST5-ECVI Predominance in a Heavily Contaminated Cheese Processing Environment	Austria	2010-2012	E
Nowak et al. (2017)	Persistent *Listeria monocytogenes* strains isolated from mussel production facilities form more biofilm but are not linked to specific genetic markers	New Zealand	1992-2011	FE
Nwaiwu et al. (2017)	Draft Genome Sequences of *Listeria monocytogenes*, Isolated from Fresh Leaf Vegetables in Owerri City, Nigeria	Nigeria	2017	F
Orsini et al. (2018)	Whole-Genome Sequences of Two *Listeria monocytogenes* Serovar 1/2a Strains Responsible for a Severe Listeriosis Outbreak in Central Italy	Italy	2015-2016	C
Ortiz et al. (2016)	The Connection between Persistent, Disinfectant-Resistant *Listeria monocytogenes* Strains from Two Geographically Separate Iberian Pork Processing Plants: Evidence from Comparative Genome Analysis	Spain	2008, 2010	E
Pasquali et al. (2018)	*Listeria monocytogenes* Sequence Types 121 and 14 Repeatedly Isolated Within One Year of Sampling in a Rabbit Meat Processing Plant: Persistence and Ecophysiology	China, France, Germany, Italy, Poland, Spain, UK, USA	2004-2017	FCE
Pirone-Davies et al. (2018)	Genes significantly associated with lineage II food isolates of *Listeria monocytogenes*	Chile, Germany, UK, USA	1975-2015	FCE
Reimer et al. (2019)	Shared genome analyses of notable listeriosis outbreaks, highlighting the critical importance of epidemiological evidence, input datasets and interpretation criteria	Canada	1981-2011	FCE
Rychli et al. (2017)	Comparative Genomics of Human and Non-Human *Listeria monocytogenes* sequence type 121 strains	Australia, Austria, China, Denmark, Egypt, France, Germany, Ireland, Italy, Rep. of Moldavia, Romania, Russia, Spain, Switzerland, Turkey, USA	1968-2015	FCE
Schjorring et al. (2017)	Cross-border outbreak of listeriosis caused by cold-smoked salmon, revealed by integrated surveillance and whole genome sequencing (WGS), Denmark and France, 2015 to 2017	Denmark, France	2015-2017	F
Stasiewicz et al. (2015)	Whole-Genome Sequencing Allows for Improved Identification of Persistent *Listeria monocytogenes* in Food-Associated Environments	USA	2001-2011	E
Tasara et al. (2016)	Genome Sequences of *Listeria monocytogenes* Strains Responsible for Cheese- and Cooked Ham Product-Associated Swiss Listeriosis Outbreaks in 2005 and 2011	Switzerland	2005, 2011	C
Toledo et al. (2018)	Genomic Diversity of *Listeria monocytogenes* Isolated from Clinical and Non-Clinical Samples in Chile	Chile	2008-2011	FCE
Zhang et al. (2016)	Evolution and Diversity of *Listeria monocytogenes* from Clinical and Food Samples in Shanghai, China	China	2004-2012	FC

REFERENCES

- Allam, M., Tau, N., Smouse, S.L., Mtshali, P.S., Mnyameni, F., Khumalo, Z.T.H., Ismail, A., Govender, N., Thomas, J., Smith, A.M., 2018. Whole-Genome Sequences of Listeria monocytogenes Sequence Type 6 Isolates Associated with a Large Foodborne Outbreak in South Africa, 2017 to 2018. Genome Announc 6.
- Bergholz, T.M., Shah, M.K., Burall, L.S., Rakic-Martinez, M., Datta, A.R., 2018. Genomic and phenotypic diversity of *Listeria monocytogenes* clonal complexes associated with human listeriosis. Appl Microbiol Biotechnol 102, 3475-3485.
- Burall, L.S., Grim, C.J., Datta, A.R., 2017. A clade of Listeria monocytogenes serotype 4b variant strains linked to recent listeriosis outbreaks associated with produce from a defined geographic region in the US. Plos One 12, e0176912.
- Chen, P., den Bakker, H.C., Korlach, J., Kong, N., Storey, D.B., Paxinos, E.E., Ashby, M., Clark, T., Luong, K., Wiedmann, M., Weimer, B.C., 2017. Comparative Genomics Reveals the Diversity of Restriction-Modification Systems and DNA Methylation Sites in Listeria monocytogenes. Appl Environ Microbiol 83.
- Chiara, M., D'Erchia, A.M., Manzari, C., Minotto, A., Montagna, C., Addante, N., Santagada, G., Latorre, L., Pesole, G., Horner, D.S., Parisi, A., 2014. Draft Genome Sequences of Six *Listeria monocytogenes* Strains Isolated from Dairy Products from a Processing Plant in Southern Italy. Genome Announc 2.
- Fagerlund, A., Langsrud, S., Schirmer, B.C.T., Møretrø, T., Heir, E., 2016. Genome Analysis of *Listeria monocytogenes* Sequence Type 8 Strains Persisting in Salmon and Poultry Processing Environments and Comparison with Related Strains. Plos One 11, e0151117.
- Fox, E.M., Allnutt, T., Bradbury, M.I., Fanning, S., Chandry, P.S., 2016. Comparative Genomics of the *Listeria monocytogenes* ST204 Subgroup. Front Microbiol 7, 2057.
- Halbedel, S., Prager, R., Fuchs, S., Trost, E., Werner, G., Flieger, A., 2018. Whole-Genome Sequencing of Recent *Listeria monocytogenes* Isolates from Germany Reveals Population Structure and Disease Clusters. J Clin Microbiol 56.
- Hilliard, A., Leong, D., O'Callaghan, A., Culligan, E.P., Morgan, C.A., DeLappe, N., Hill, C., Jordan, K., Cormican, M., Gahan, C.G.M., 2018. Genomic Characterization of Listeria monocytogenes Isolates Associated with Clinical Listeriosis and the Food Production Environment in Ireland. Genes (Basel) 9.
- Hingston, P., Chen, J., Dhillon, B.K., Laing, C., Bertelli, C., Gannon, V., Tasara, T., Allen, K., Brinkman, F.S., Truelstrup Hansen, L., Wang, S., 2017. Genotypes Associated with *Listeria monocytogenes* Isolates Displaying Impaired or Enhanced Tolerances to Cold, Salt, Acid, or Desiccation Stress. Front Microbiol 8, 369.
- Horlbog, J.A., Jang, H., Gopinath, G., Stephan, R., Guldimann, C., 2018. Whole-Genome Sequences of Six Listeria monocytogenes Strains Isolated from Food. Microbiol Resour Announc 7.
- Hyden, P., Pietzka, A., Lennkh, A., Murer, A., Springer, B., Blaschitz, M., Indra, A., Huhulescu, S., Allerberger, F., Ruppitsch, W., Sensen, C.W., 2016. Whole genome sequence-based serogrouping of Listeria monocytogenes isolates. J Biotechnol 235, 181-186.
- Kim, S.W., Haendiges, J., Keller, E.N., Myers, R., Kim, A., Lombard, J.E., Karns, J.S., Van Kessel, J.A.S., Haley, B.J., 2018. Genetic diversity and virulence profiles of Listeria monocytogenes recovered from bulk tank milk, milk filters, and milking equipment from dairies in the United States (2002 to 2014). Plos One 13, e0197053.
- Kwong, J.C., Mercoulia, K., Tomita, T., Easton, M., Li, H.Y., Bulach, D.M., Stinear, T.P., Seemann, T., Howden, B.P., 2016. Prospective Whole-Genome Sequencing Enhances National Surveillance of *Listeria monocytogenes*. J Clin Microbiol 54, 333-342.
- Lomonaco, S., Nucera, D., Filipello, V., 2015. The evolution and epidemiology of Listeria monocytogenes in Europe and the United States. Infect Genet Evol 35, 172-183.
- Lopez-Alonso, V., Ortiz, S., Martinez-Suarez, J.V., 2019. Whole-Genome Sequences of Seven Listeria monocytogenes Strains from Different Stages of a Poultry Meat Production Chain. Microbiol Resour Announc 8.
- Maury, M.M., Tsai, Y.H., Charlier, C., Touchon, M., Chenal-Francisque, V., Leclercq, A., Criscuolo, A., Gaultier, C., Roussel, S., Brisabois, A., Disson, O., Rocha, E.P.C., Brisse, S., Lecuit, M., 2016. Uncovering *Listeria monocytogenes* hypervirulence by harnessing its biodiversity. Nature Genetics 48, 308-313.
- Morganti, M., Scaltriti, E., Cozzolino, P., Bolzoni, L., Casadei, G., Pierantoni, M., Foni, E., Pongolini, S., 2016. Processing-Dependent and Clonal Contamination Patterns of *Listeria monocytogenes* in the Cured

Ham Food Chain Revealed by Genetic Analysis. Appl Environ Microbiol 82, 822-831.

- Moura, A., Criscuolo, A., Pouseele, H., Maury, M.M., Leclercq, A., Tarr, C., Bjorkman, J.T., Dallman, T., Reimer, A., Enouf, V., Larsonneur, E., Carleton, H., Bracq-Dieye, H., Katz, L.S., Jones, L., Touchon, M., Tourdjman, M., Walker, M., Stroika, S., Cantinelli, T., Chenal-Francisque, V., Kucerova, Z., Rocha, E.P.C., Nadon, C., Grant, K., Nielsen, E.M., Pot, B., Gerner-Smidt, P., Lecuit, M., Brisse, S., 2017. Whole genome-based population biology and epidemiological surveillance of *Listeria monocytogenes*. Nature Microbiology 2, 16185.
- Muhterem-Uyar, M., Ciolacu, L., Wagner, K.H., Wagner, M., Schmitz-Esser, S., Stessl, B., 2018. New Aspects on *Listeria monocytogenes* ST5-ECVI Predominance in a Heavily Contaminated Cheese Processing Environment. Front Microbiol 9, 64.
- Nowak, J., Cruz, C.D., Tempelaars, M., Abee, T., van Vliet, A.H.M., Fletcher, G.C., Hedderley, D., Palmer, J., Flint, S., 2017. Persistent Listeria monocytogenes strains isolated from mussel production facilities form more biofilm but are not linked to specific genetic markers. International Journal of Food Microbiology 256, 45-53.
- Nwaiwu, O., Moura, A., Thouvenot, P., Rees, C., Leclercq, A., Lecuit, M., 2017. Draft Genome Sequences of Listeria monocytogenes, Isolated from Fresh Leaf Vegetables in Owerri City, Nigeria. Genome Announc 5.
- Orsini, M., Cornacchia, A., Patavino, C., Torresi, M., Centorame, P., Acciari, V.A., Ruolo, A., Marcacci, M., Ancora, M., Di Domenico, M., Mangone, I., Blasi, G., Duranti, A., Camma, C., Pomilio, F., Migliorati, G., 2018. Whole-Genome Sequences of Two Listeria monocytogenes Serovar 1/2a Strains Responsible for a Severe Listeriosis Outbreak in Central Italy. Genome Announc 6.
- Ortiz, S., Lopez-Alonso, V., Rodriguez, P., Martinez-Suarez, J.V., 2016. The Connection between Persistent, Disinfectant-Resistant *Listeria monocytogenes* Strains from Two Geographically Separate Iberian Pork Processing Plants: Evidence from Comparative Genome Analysis. Appl Environ Microbiol 82, 308-317.
- Pasquali, F., Palma, F., Guillier, L., Lucchi, A., De Cesare, A., Manfreda, G., 2018. *Listeria monocytogenes* Sequence Types 121 and 14 Repeatedly Isolated Within One Year of Sampling in a Rabbit Meat Processing Plant: Persistence and Ecophysiology. Front Microbiol 9, 596.
- Pirone-Davies, C., Chen, Y., Pightling, A., Ryan, G., Wang, Y., Yao, K., Hoffmann, M., Allard, M.W., 2018. Genes significantly associated with lineage II food isolates of Listeria monocytogenes. Bmc Genomics 19, 708.
- Reimer, A., Weedmark, K., Petkau, A., Peterson, C.L., Walker, M., Knox, N., Kent, H., Mabon, P., Berry, C., Tyler, S., Tschetter, L., Jerome, M., Allen, V., Hoang, L., Bekal, S., Clark, C., Nadon, C., Van Domselaar, G., Pagotto, F., Graham, M., Farber, J., Gilmour, M., 2019. Shared genome analyses of notable listeriosis outbreaks, highlighting the critical importance of epidemiological evidence, input datasets and interpretation criteria. Microb Genom 5.
- Rychli, K., Wagner, E.M., Ciolacu, L., Zaiser, A., Tasara, T., Wagner, M., Schmitz-Esser, S., 2017. Comparative genomics of human and non-human *Listeria monocytogenes* sequence type 121 strains. Plos One 12, e0176857.
- Schjorring, S., Gillesberg Lassen, S., Jensen, T., Moura, A., Kjeldgaard, J.S., Muller, L., Thielke, S., Leclercq, A., Maury, M.M., Tourdjman, M., Donguy, M.P., Lecuit, M., Ethelberg, S., Nielsen, E.M., 2017. Cross-border outbreak of listeriosis caused by cold-smoked salmon, revealed by integrated surveillance and whole genome sequencing (WGS), Denmark and France, 2015 to 2017. Euro Surveill 22.
- Stasiewicz, M.J., Oliver, H.F., Wiedmann, M., den Bakker, H.C., 2015. Whole-Genome Sequencing Allows for Improved Identification of Persistent *Listeria monocytogenes* in Food-Associated Environments. Appl Environ Microbiol 81, 6024-6037.
- Tasara, T., Klumpp, J., Bille, J., Stephan, R., 2016. Genome Sequences of Listeria monocytogenes Strains Responsible for Cheese- and Cooked Ham Product-Associated Swiss Listeriosis Outbreaks in 2005 and 2011. Genome Announc 4.
- Toledo, V., den Bakker, H.C., Hormazabal, J.C., Gonzalez-Rocha, G., Bello-Toledo, H., Toro, M., Moreno-Switt, A.I., 2018. Genomic Diversity of Listeria monocytogenes Isolated from Clinical and Non-Clinical Samples in Chile. Genes (Basel) 9.
- Zhang, J., Cao, G., Xu, X., Allard, M., Li, P., Brown, E., Yang, X., Pan, H., Meng, J., 2016. Evolution and Diversity of Listeria monocytogenes from Clinical and Food Samples in Shanghai, China. Frontiers in Microbiology 7, 1138.
