# Supplementary material for: A Large-Scale Sequencing-Based Survey of Plasmids in Listeria monocytogenes Reveals Global Dissemination of Plasmids
Source: Front Microbiol. 2021 Mar 12;12:653155. doi: 10.3389/fmicb.2021.653155 (PMC7994336; doi:10.3389/fmicb.2021.653155)
Supplement: Supplementary file 11 [file Data_Sheet_11.PDF]

Supplementary Table 3. Selected *L. monocytogenes* plasmids used as queries for Blastn analysis for determination of plasmid presence and contigs. Source types are F: food, C: clinical, E: Environmental, U: unknown. The detailed references can be found below the Table.

**ST**	**Reference strain** (if not indicated otherwise, all strains are *L. monocytogenes*)	**Plasmid**	**Plasmid size (bp)**	**Country of origin**	**Source / year**	**Accession number**	**Reference**	**RepA group**
3	N1-011a	pLMN1-011a	148,959	USA	E / 2013	CP006611	unpublished	Group 1 and 2
6	H7858	pLM80	82,248	USA	F / 1998	AADR01	(Nelson et al., 2004)	Group 2
7	2015TE24968	pl2015TE24968	57,530	Italy	C / 2015	NZ_CP015985	(Orsini et al., 2018)	Group 1
8	R479a	pLMR479a	86,652	Denmark	F / 1996	HG813248	(Schmitz-Esser, Gram, et al., 2015)	Group 2
9	AT3E	pLM58	58,523	Finland	F / 1995	NZ_CP023753	(Pontinen et al., 2017)	Group 1
14	LM1	pLM33	32,307	Spain	F / 1992	GU244485	(Canchaya et al., 2010)	Group 1
120	08-5578	pLM5578	77,054	Canada	F / 2008	NC_013767	Gilmour et al. (2010)	Group 2
121	6179	pLM6179	62,206	Ireland	F / 2000	HG813250	(Schmitz-Esser, Muller, et al., 2015)	Group 2
3	SLCC2482	pLM7UG1	50,100	UK	C / 1966	FR667690	(Kuenne et al., 2010)	Group 1
66	SLCC2755	pLM1-2bUG1	57,780	UK	C / 1967	FR667692	(Kuenne et al., 2010)	Group 1
6	ST6	pLMST6	4,268	Netherlands	C / 2017	LT732640	Kremer et al. (2017)	n/a*
	BM4293	pIP823	3,712	France	E	U40997	(Charpentier et al., 1999)	n/a*
	L. grayi DSM20601	pLGUG1	79,249		C	FR667693	(Kuenne et al., 2010)	Group 2
	L. welshimeri 40/07	pLIS1	81,588	Poland	F / 2007	MH382833	(Korsak et al., 2019)	Group 2

* as the small *Listeria* plasmids have plasmid replication proteins that are unrelated to the group 1 and 2 RepA proteins, these small plasmids cannot be grouped into these RepA groups.

REFERENCES

- Canchaya, C., Giubellini, V., Ventura, M., de los Reyes-Gavilan, C.G., Margolles, A., 2010. Mosaic-like sequences containing transposon, phage, and plasmid elements among *Listeria monocytogenes* plasmids. Appl Environ Microbiol 76, 4851-4857.
- Charpentier, E., Gerbaud, G., Courvalin, P., 1999. Conjugative mobilization of the rolling-circle plasmid pIP823 from *Listeria monocytogenes* BM4293 among gram-positive and gram-negative bacteria. J Bacteriol 181, 3368-3374.
- Gilmour, M.W., Graham, M., Van Domselaar, G., Tyler, S., Kent, H., Trout-Yakel, K.M., Larios, O., Allen, V., Lee, B., Nadon, C., 2010. High-throughput genome sequencing of two Listeria monocytogenes clinical isolates during a large foodborne outbreak. Bmc Genomics 11, 120.
- Korsak, D., Chmielowska, C., Szuplewska, M., Bartosik, D., 2019. Prevalence of plasmid-borne benzalkonium chloride resistance cassette *bcrABC* and cadmium resistance *cadA* genes in nonpathogenic *Listeria* spp. isolated from food and food-processing environments. Int J Food Microbiol 290, 247-253.
- Kremer, P.H.C., Lees, J.A., Koopmans, M.M., Ferwerda, B., Arends, A.W.M., Feller, M.M., Schipper, K., Seron, M.V., van der Ende, A., Brouwer, M.C., van de Beek, D., Bentley, S.D., 2017. Benzalkonium tolerance genes and outcome in *Listeria monocytogenes* meningitis. Clinical Microbiology and Infection 23, 265e261-265e267.
- Kuenne, C., Voget, S., Pischimarov, J., Oehm, S., Goesmann, A., Daniel, R., Hain, T., Chakraborty, T., 2010. Comparative analysis of plasmids in the genus *Listeria*. Plos One 5.
- Nelson, K.E., Fouts, D.E., Mongodin, E.F., Ravel, J., DeBoy, R.T., Kolonay, J.F., Rasko, D.A., Angiuoli, S.V., Gill, S.R., Paulsen, I.T., Peterson, J., White, O., Nelson, W.C., Nierman, W., Beanan, M.J., Brinkac, L.M., Daugherty, S.C., Dodson, R.J., Durkin, A.S., Madupu, R., Haft, D.H., Selengut, J., Van Aken, S., Khouri, H., Fedorova, N., Forberger, H., Tran, B., Kathariou, S., Wonderling, L.D., Uhlich, G.A., Bayles, D.O., Luchansky, J.B., Fraser, C.M., 2004. Whole genome comparisons of serotype 4b and 1/2a strains of the food-borne pathogen *Listeria monocytogenes* reveal new insights into the core genome components of this species. Nucleic Acids Res 32, 2386-2395.
- Orsini, M., Cornacchia, A., Patavino, C., Torresi, M., Centorame, P., Acciari, V.A., Ruolo, A., Marcacci, M., Ancora, M., Di Domenico, M., Mangone, I., Blasi, G., Duranti, A., Camma, C., Pomilio, F., Migliorati, G., 2018. Whole-Genome Sequences of Two Listeria monocytogenes Serovar 1/2a Strains Responsible for a Severe Listeriosis Outbreak in Central Italy. Genome Announc 6.
- Pontinen, A., Aalto-Araneda, M., Lindstrom, M., Korkeala, H., 2017. Heat Resistance Mediated by pLM58 Plasmid-Borne ClpL in *Listeria monocytogenes*. mSphere 2.
- Schmitz-Esser, S., Gram, L., Wagner, M., 2015. Complete Genome Sequence of the Persistent *Listeria monocytogenes* Strain R479a. Genome Announc 3, e00150-00115.
- Schmitz-Esser, S., Muller, A., Stessl, B., Wagner, M., 2015. Genomes of sequence type 121 *Listeria monocytogenes* strains harbor highly conserved plasmids and prophages. Front Microbiol 6, 380.
